# Five New Iridoids From the Leaves of *Viburnum macrocephalum* f*. macrocephalum* and Their Free Radical Scavenging Activity

**DOI:** 10.1002/cbdv.71649

**Published:** 2026-09-03

**Authors:** Ya‐Jiao Chen, Hong‐Juan Zhou, Jia Chen, Jian‐Hua Shao, Chun‐Chao Zhao

**Affiliations:** ^1^ College of Bioscience and Biotechnology Yangzhou University Yangzhou China

**Keywords:** ABTS, DPPH, iridoid, *Viburnum macrocephalum* f. *macrocephalum*

## Abstract

An investigation of the chemical constituents of *Viburnum macrocephalum* f. *macrocephalum* leaves (VMML) resulted in the purification of five previously unreported iridoids, viburetins D–H (**1**–**5**), along with one known analogue (**6**). Compounds **1**–**5** were identified by combined spectroscopic and spectrometric techniques, including 1D/2D NMR, HRESIMS, and ECD. Antioxidant assays revealed that compounds **2**, **5**, and **6** exhibited DPPH (IC_50_: 36.58–53.90 µM) and ABTS (IC_50_: 25.96–45.80 µM) free radical scavenging activity, whereas compound **1** was appreciably active in the ABTS assay (IC_50_ = 58.47 µM). Notably, compound **6** was the most potent antioxidant, showing greater activity than the positive control Trolox in both assays (DPPH: IC_50_ = 36.58 vs. 41.12 µM; ABTS: IC_50_ = 25.96 vs. 29.09 µM). These findings have enriched the chemical diversity of iridoids from VMML and provided preliminary evidence for their free radical scavenging activity.

## Graphical abstract

Five new iridoids, viburetins D–H, featuring dioxatricyclodecane and ring‑opened monocyclic skeletons, are isolated from the leaves of *Viburnum macrocephalum* f. *macrocephalum*, enriching the chemical diversity of *Viburnum*. Preliminary DPPH and ABTS assays reveal varying degrees of radical scavenging activity.

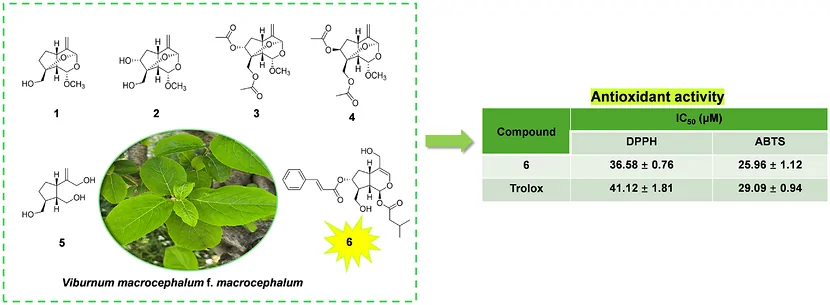

## 1 Introduction

Iridoids constitute a structurally diverse and biologically significant class of bicyclic monoterpenes derived biosynthetically from iridodial. The first iridoid, iridomyrmecin, was identified in 1925 from the ant *Iridomyrmex humilis*. Iridoids are universally characterized by a cyclopenta[*c*]pyran skeleton [1]. Recently, iridoids have attracted increasing attention due to their antimicrobial [2], antiviral [3], anti‐inflammatory [4], and antioxidant activities [5]. Furthermore, studies have demonstrated that plant‐derived iridoid glycosides are effective in treating various diseases, including liver diseases [6], cardiovascular diseases [7], and tumors [8].

**FIGURE 1:**
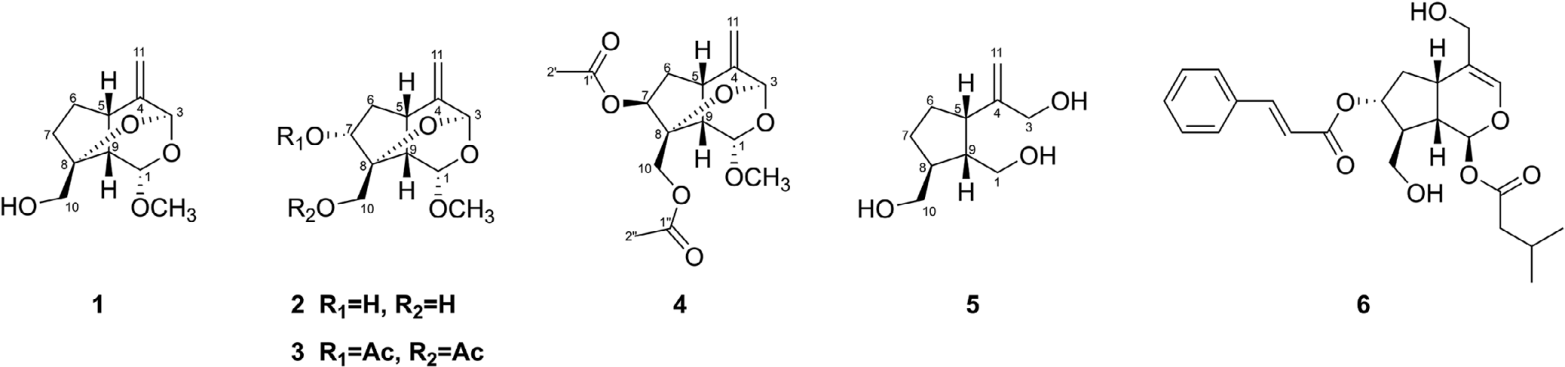
Chemical structures of compounds **1**–**6**.

The genus *Viburnum* (Adoxaceae), comprising over 230 species of shrubs and small trees, is widely distributed in the Northern Hemisphere. Phytochemical studies have revealed these plants to be rich sources of structurally diverse secondary metabolites, including monoterpenes, sesquiterpenes, diterpenoids, and lignans, many of which exhibit potent anti‐inflammatory, antitumor, and antioxidant activities [9]. *Viburnum macrocephalum* f. *macrocephalum* (VMM), native to China and commonly known as “Muxiuqiu” or Chinese snowball viburnum, is extensively cultivated in several provinces, including Jiangsu, Zhejiang, Jiangxi, and Hebei. In traditional Chinese medicine, the stems and leaves of VMM are used to eliminate dampness and relieve pruritus, and are applied in the treatment of scabies, tinea, and eczematous itching [10]. Recent phytochemical and pharmacological studies have demonstrated that the flowers of VMM possess antioxidant, antibacterial, and antiviral properties [11, 12]. Nevertheless, the leaf metabolites of VMM remain largely uncharacterized. As part of our continuing interest in bioactive natural products from *Viburnum*, we conducted a phytochemical investigation of *V*. *macrocephalum* f. *macrocephalum* leaves (VMML). The isolation, structural characterization, and DPPH/ABTS antioxidant evaluation of five new iridoids (viburetins D–H, **1**–**5**) and one known compound (**6**) are reported here (Figure 1).

## 2 Results and Discussion

The extract from VMML was fractionated through a series of chromatographic separations to obtain five new iridoids, named viburetins D–H (**1**–**5**), and the known iridoid viburnshosin D (**6**) (Figure 1).

**FIGURE 2:**
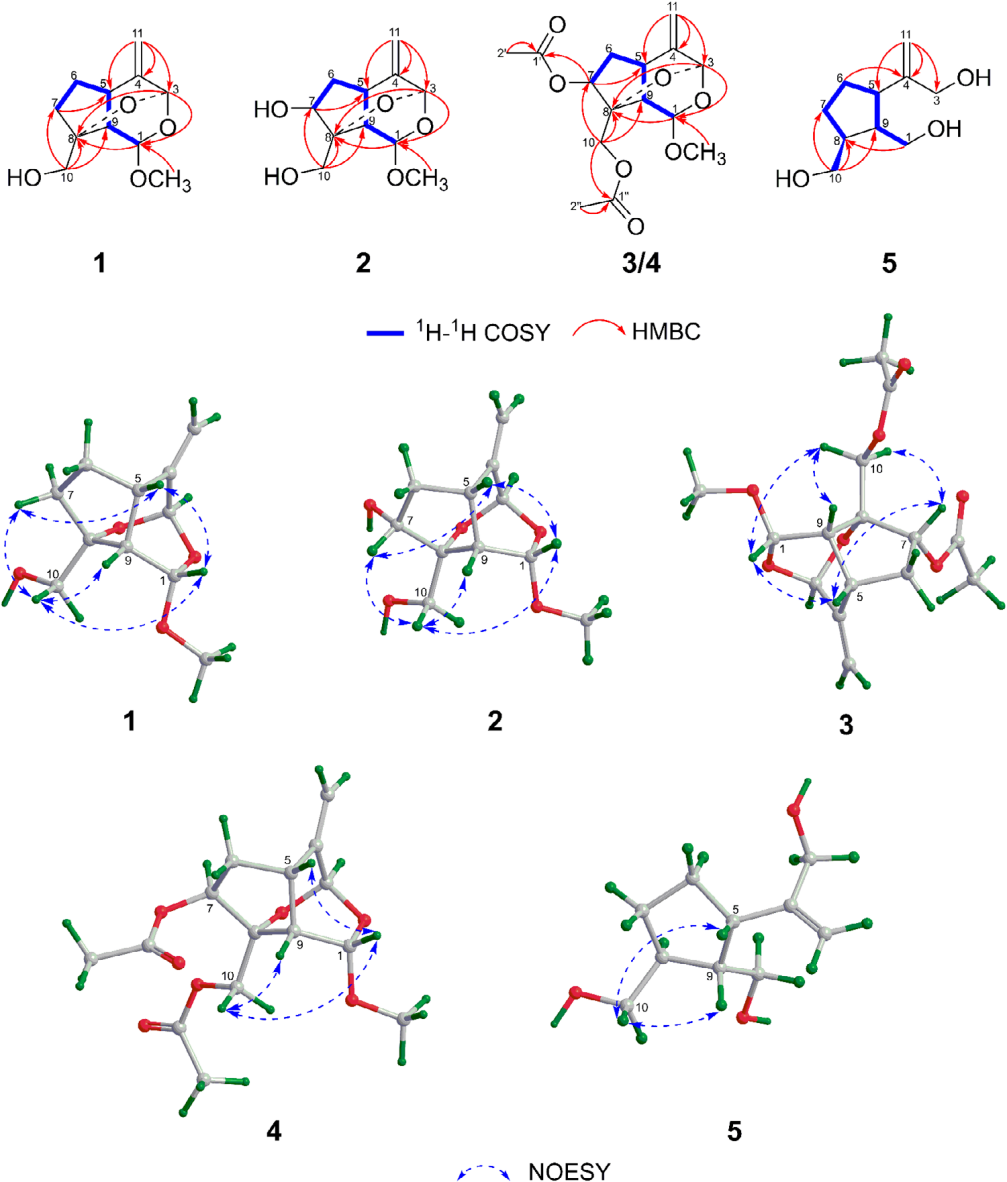
Key 2D NMR correlations of compounds **1**–**5**.

**FIGURE 3:**
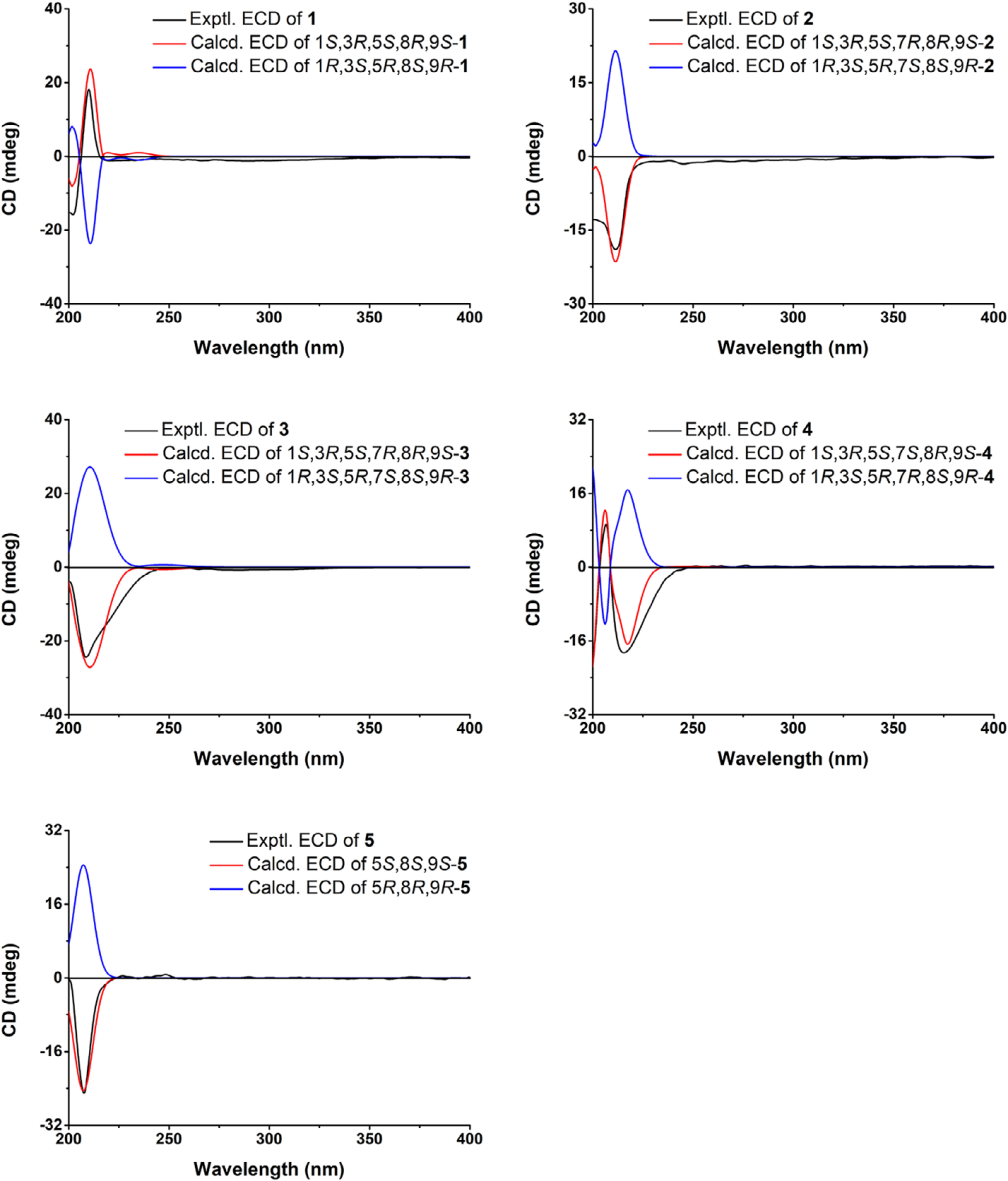
Experimental and calculated ECD spectra of compounds **1**–**5**.

Viburetin D (**1**) was isolated as a colorless oil. Its molecular formula was determined to be C_11_H_16_O_4_ by HRESIMS data, which showed a sodium adduct at *m/z* 235.0939 [M+Na]^+^ (calcd for C_11_H_16_O_4_Na^+^, 235.0941), indicating four degrees of unsaturation. The ^1^H and ^13^C NMR spectra (Tables 1 and 2) displayed the signals characteristic of two acetal methines [*δ*_H_ 5.04 (d, *J* = 3.0 Hz), *δ*_C_ 98.3; *δ*_H_ 5.03 (s), *δ*_C_ 94.1], one oxygenated methylene [*δ*_H_ 3.62 (d, *J* = 11.6 Hz), 3.59 (d, *J* = 11.6 Hz), *δ*_C_ 64.9], one methoxy group [*δ*_H_ 3.35 (s), *δ*_C_ 55.3], one exocyclic methylene [*δ*_H_ 4.89 (d, *J* = 1.6 Hz), 4.81 (d, *J* = 1.6 Hz), *δ*_C_ 107.9], one oxygenated quaternary carbon (*δ*_C_ 84.6), and two aliphatic methines, together with two aliphatic methylenes. The ^1^H–^1^H COSY spectrum revealed a spin system corresponding to the H‐1/H‐9/H‐5/H_2_‐6/H_2_‐7 fragment (Figure 2). HMBC cross‐peaks (Figure 2) from OCH_3_ to C‐1 located the methoxy group at this position, while correlations of H_2_‐11 with C‐3, C‐4 (*δ*_C_ 151.8), and C‐5 (*δ*_C_ 39.8) defined the C‐4/C‐11 double bond; additionally, H_2_‐10 showed HMBC contacts to C‐7 (*δ*_C_ 34.7), C‐8, and C‐9 (*δ*_C_ 43.9), placing the hydroxymethyl substituent at C‐8. The remaining H‐3 → C‐8 correlation, taken together with the four degrees of unsaturation of compound **1**, supported the presence of a 3,8‐ether bridge. Thus, the planar structure of compound **1** was elucidated as an iridoid featuring a dioxatricyclodecane skeleton [13, 14]. Regarding the relative configuration, the conventional *β*‐orientation of H‐5 and H‐9 is common in iridoids from *Viburnum* plants, and the small coupling constant (*J* = 3.0 Hz) between H‐1 and H‐9 supported the assignment of a *β*‐configuration for H‐1 [15]. The relative configuration of compound **1** was further confirmed by NOESY correlations of H_2_‐10 with H‐1, H‐9, and H‐7b (*δ*_H_ 1.63), and of H‐7b with H‐5 (Figure 2), indicating the *β*‐orientation of H‐1, H‐5, and H‐9. The absolute configuration of **1** was unambiguously established as 1*S*,3*R*,5*S*,8*R*,9*S* by the comparison between its experimental and calculated ECD spectra (Figure 3). Based on the above evidence and a search of the SciFinder database, the structure of **1** was identified as a new compound and was named viburetin D.

**TABLE 1:** ^1^H NMR data of compounds **1**–**5** (600 MHz, *δ* in ppm, *J* in Hz).

	1^a^	2^b^	3^a^	4^a^	5^c^
1	5.04 (d, 3.0)	5.01 (d, 3.1)	5.05 (d, 3.2)	5.00 (d, 3.0)	3.24, dd (10.1, 5.0) 3.00, dd (10.1, 5.6)
3	5.03 (s)	5.09 (s)	5.10 (s)	5.10 (s)	3.90, dd (14.5, 5.3) 3.82, dd (14.5, 5.7)
5	3.11–3.14 (m)	3.18–3.20 (m)	3.20–3.23 (m)	3.09–3.12 (m)	2.41–2.45, m
6a	1.95–1.97 (m)	2.15 (dd, 13.8, 7.3)	2.15 (dd, 14.4, 7.5)	2.22 (dd, 14.2, 7.4)	1.61–1.64, m
6b	1.48–1.53 (m)	1.84 (dd, 13.8, 7.2)	1.93 (ddd, 14.4, 7.4, 3.0)	1.95 (ddd, 14.2, 7.2, 3.4)	1.55–1.58, m
7a	1.97–2.04 (m)	4.19 (dd, 7.3, 3.4)	5.01 (dd, 7.5, 3.0)	5.01 (dd, 7.4, 3.4)	1.75–1.78, m
7b	1.60–1.67 (m)				1.22–1.25, m
8					1.96–1.99, m
9	2.20 (dd, 4.6, 3.0)	2.48 (dd, 4.8, 3.1)	2.56 (dd, 5.0, 3.2)	2.36 (dd, 5.1, 3.0)	1.82–1.85, m
10a	3.62 (d, 11.6)	3.94 (d, 11.7)	4.44 (d, 11.7)	4.63 (d, 11.9)	3.34, ddd (9.8, 6.7, 4.8)
10b	3.59 (d, 11.6)	3.86 (d, 11.7)	4.26 (d, 11.7)	4.32 (d, 11.9)	3.27–3.30, m
11a	4.89 (d, 1.6)	4.91 (d, 1.5)	4.94 (d, 1.5)	4.99 (d, 1.4)	5.02, d (1.5)
11b	4.81 (d, 1.6)	4.83 (d, 1.5)	4.82 (d, 1.5)	4.91 (d, 1.4)	4.73, d (1.5)
1‐OCH_3_	3.35 (s)	3.39 (s)	3.36 (s)	3.44 (s)	—
7‐COCH_3_	—	—	2.00 (s)	1.97 (s)	—
10‐COCH_3_	—	—	2.04 (s)	1.99 (s)	—

^a^Measured in CD_3_OD.

^b^Measured in CDCl_3._

^c^Measured in DMSO‐*d*_6_.

**TABLE 2:** ^13^C NMR data of compounds **1**–**5** (150 MHz, *δ* in ppm).

	1^a^	2^b^	3^a^	4^a^	5^c^
1	98.3	96.9	97.5	99.3	61.9
3	94.1	93.7	94.6	94.1	64.8
4	151.8	148.8	150.5	149.5	149.9
5	39.8	36.5	38.0	40.5	43.5
6	31.4	42.2	41.6	41.8	28.6
7	34.7	78.1	79.1	78.9	27.4
8	84.6	83.6	82.9	83.3	44.7
9	43.9	40.8	43.0	43.4	45.0
10	64.9	64.2	65.6	67.6	65.5
11	107.9	108.0	108.2	109.1	107.4
1‐OCH_3_	55.3	55.4	55.2	56.2	—
7‐COCH_3_	—	—	171.4	171.2	—
7‐COCH_3_	—	—	20.9	20.9	—
10‐COCH_3_	—	—	172.3	172.4	—
10‐COCH_3_	—	—	20.7	20.6	—

^a^Measured in CD_3_OD.

^b^Measured in CDCl_3._

^c^Measured in DMSO‐*d*_6_.

Viburetin E (**2**), obtained as a colorless oil, was assigned the molecular formula C_11_H_16_O_5_ based on the HRESIMS ion peak at *m*/*z* 251.0886 [M+Na]^+^ (calcd for C_11_H_16_O_5_Na^+^, 251.0890), implying four indices of hydrogen deficiency. The 1D NMR spectra of **2** (Tables 1 and 2) closely resembled those of chlorovaltrate R [16], consistent with a shared iridoid framework. The primary difference was observed at C‐10 (*δ*_C_ 78.1) of **2**, whose signal exhibited a downfield shift of 1.0 ppm relative to that of chlorovaltrate R, indicating the replacement of the C‐10 chlorine substituent by a hydroxyl group. The assignment was supported by HMBC correlations from H‐5 (*δ*_H_ 3.19) and H_2_‐10 (*δ*_H_ 3.94 and 3.86) to C‐7 (Figure 2). The relative configurations of H‐1/H‐5/H‐9 were determined to be identical to those of **1** based on biosynthetic grounds and NOESY correlations (Figure 2). Key NOESY cross‐peaks from H‐7 (*δ*_H_ 4.19) to H_2_‐10 suggested a *β*‐orientation for H‐7. The absolute configuration of **2** was unambiguously assigned as 1*S*,3*R*,5*S*,7*R*,8*R*,9*S*, based on the close agreement between its experimental and calculated ECD spectra (Figure 3). Drawing on the above evidence and the results of a comprehensive SciFinder search, the structure of **2** was established as a new iridoid, named viburetin E.

The molecular formulas of viburetins F (**3**) and G (**4**) were determined to be C_15_H_20_O_7_, based on their respective [M+Na]^+^ peaks at *m*/*z* 335.1112 and 335.1108 (calcd for C_15_H_20_O_7_Na^+^, 335.1101). These formulas corresponded to six degrees of unsaturation. The 1D NMR spectra of **3** and **4** (Tables 1 and 2) displayed highly similar signal patterns, suggesting that they were a pair of stereoisomers. Comparison of their 1D NMR data with those of **2** revealed that they possessed an identical iridoid skeleton, characterized by two acetal methines, one oxygenated methylene, two aliphatic methines, and one exocyclic methylene group. The primary difference lay in the presence of two additional acetoxy groups in **3** and **4** compared to **2**. The placements of the acetoxy groups at C‐7 and C‐10 in **3** and **4** were confirmed by HMBC correlations from H‐7 to one ester carbonyl carbon (C‐1′) and from H_2_‐10 to the other ester carbonyl carbon (C‐1′′), respectively (Figure 2). In compound **3**, key NOESY correlations observed between H_2_‐10 (*δ*_H_ 4.44 and 4.26) and H‐1 (*δ*_H_ 5.05), H‐7 (*δ*_H_ 5.01), and H‐9 (*δ*_H_ 2.56), as well as between H‐5 (*δ*_H_ 3.21) and H‐1/H‐7, indicated that H‐1, H‐5, H‐7, and H‐9 were all *β*‐oriented. In contrast, the NOESY spectrum of **4** showed correlations from H_2_‐10 (*δ*_H_ 4.63 and 4.32) to H‐5 (*δ*_H_ 3.10) and H‐9 (*δ*_H_ 2.36), and from H‐5 to H‐1 (*δ*_H_ 5.00), confirming the *β*‐orientation of H‐1, H‐5, and H‐9. The identical configurations at all positions except C‐7, together with the lack of NOESY correlations between H‐7 and H_2_‐10, indicated an *α*‐orientation for H‐7 in **4**. The experimental ECD spectrum of **3** was in good agreement with the calculated spectrum for the (1*S*,3*R*,5*S*,7*R*,8*R*,9*S*) configuration, whereas that of **4** corresponded to the (1*S*,3*R*,5*S*,7*S*,8*R*,9*S*) stereoisomer (Figure 3). SciFinder searches revealed no prior reports of either compound. Compounds **3** and **4** were therefore characterized as new iridoids and were named viburetins F and G, respectively.

Viburetin H (**5**) was obtained as a colorless oil. Its molecular formula was determined to be C_10_H_18_O_3_ based on the HRESIMS ion peak at *m/z* 209.1148 [M+Na]^+^ (calcd for C_10_H_18_O_3_Na^+^, 209.1148), suggesting two indices of hydrogen deficiency. In the 1D NMR spectra of **5** (Tables 1 and 2), the characteristic signals were observed for three hydroxymethyl groups [*δ*_H_ 3.24 (dd, *J* = 10.1, 5.0 Hz) and 3.00 (dd, *J* = 10.1, 5.6 Hz), *δ*_C_ 61.9; *δ*_H_ 3.90 (dd, *J* = 14.5, 5.3 Hz) and 3.82 (dd, *J* = 14.5, 5.7 Hz), *δ*_C_ 64.8; *δ*_H_ 3.34 (ddd, *J* = 9.8, 6.7, 4.8 Hz) and 3.29 (m), *δ*_C_ 65.5], two aliphatic methylenes [*δ*_H_ 1.63 (m) and 1.57 (m), *δ*_C_ 28.6; *δ*_H_ 1.77 (m) and 1.23 (m), *δ*_C_ 27.4], and three aliphatic methines [*δ*_H_ 2.43 (m), *δ*_C_ 43.5; *δ*_H_ 1.98 (m), *δ*_C_ 44.7; *δ*_H_ 1.84 (m), *δ*_C_ 45.0]. Additionally, signals for one exocyclic methylene [*δ*_H_ 5.02 (d, *J* = 1.5 Hz) and 4.73 (d, *J* = 1.5 Hz), *δ*_C_ 107.4] and one quaternary carbon (*δ*_C_ 149.9) were identified. The above spectroscopic data resembled those of viburnshosin A [15], except for the chemical shifts at C‐7 and C‐10. A spin system corresponding to the H‐1/H‐9/H‐5/H_2_‐6/H_2_‐7/H‐8/H_2_‐10 fragment was established by ^1^H–^1^H COSY correlations (Figure 2). The absence of key HMBC correlations from H_2_‐1 to C‐3 indicated that compound **5** was a monocyclic iridoid derived from the opening of the 1,3‐epoxy motif in the iridoid skeleton (Figure 2). The *β*‐orientations of H‐5 and H‐9 (inferred from biosynthetic considerations) and of H_2_‐10 were established by key NOESY correlations (Figure 2) from H_2_‐10 to H‐5 and H‐9. The absolute stereochemistry of **5** was verified as 5*S*,8*S*,9*S* through the agreement observed between the experimental and calculated ECD curves (Figure 3). Based on a SciFinder search, compound **5** was identified as a new compound and named viburetin H.

Compound **6** was identified as viburnshosin D by comparison of its spectroscopic data with those reported in the literature [15].

All isolated iridoids were evaluated for their antioxidant capacity (Table 3). In the DPPH radical scavenging assay, compounds **2**, **5**, and **6** exhibited activities, with IC_50_ values ranging from 36.58 to 53.90 µM. Compound **1** showed weak activity (IC_50_ = 87.31 µM), while compounds **3** and **4** were inactive (IC_50_> 100 µM). Notably, compound **6** displayed potent activity (IC_50_ = 36.58 µM), which was superior to that of the positive control Trolox (IC_50_ = 41.12 µM), and compound **5** (IC_50_ = 43.23 µM) showed appreciable activity. In the ABTS radical scavenging assay, compounds **1**, **2**, **5**, and **6** demonstrated varying degrees of antioxidant potential, with IC_50_ values ranging from 25.96 to 58.47 µM, whereas compounds **3** and **4** remained inactive (IC_50_> 100 µM). Among them, compound **6** exhibited the most potent activity (IC_50_ = 25.96 µM), surpassing Trolox (IC_50_ = 29.09 µM), while compound **2** showed comparable potency (IC_50_ = 31.52 µM). Preliminary structure–activity relationship analysis suggested that the free radical scavenging ability of these isolates was contingent upon the presence of hydroxyl groups and an enol ether double bond. This was exemplified by compound **6**, which possessed these features and exhibited potent activity, whereas compounds **3** and **4**, which were 7,10‑diacetylated derivatives of compound **2**, exhibited markedly reduced radical‑scavenging activities. This loss of activity may be attributed to the acetylation of the C‑7 and C‑10 hydroxyls, which eliminated the hydrogen atom transfer pathway. Additionally, delocalization of the ester oxygen lone pairs into the adjacent carbonyl attenuated their single‐electron transfer capacity. Meanwhile, the potent radical‑scavenging activity of compound **6** relative to compounds **1**–**5** might be associated with the presence of the cinnamoyl ester moiety [17]. Overall, with the exception of compound **6**, the isolated iridoids (**2** and **5**) exhibited only modest radical‑scavenging activities in these assays. Although iridoids have been reported to possess antioxidant activity in various plant species [18], the antioxidant potential of the iridoid fraction from *Viburnum* species has not been previously investigated, as earlier studies on this genus have focused primarily on phenolic constituents [19]. Given that DPPH and ABTS assays provide related information on radical‑scavenging activity, future studies employing complementary methods, such as FRAP or ORAC, in conjunction with cell‐based oxidative stress models, would provide a more comprehensive antioxidant evaluation.

**TABLE 3:** Radical‐scavenging activities of iridoids **1**–**6** from VMML.

Compound	IC_50_ (µM)	
	DPPH	ABTS
**1**	87.31 ± 3.36^a^	58.47 ± 3.02^a^
**2**	53.90 ± 3.09^b^	31.52 ± 1.46^c^
**3**	> 100	> 100
**4**	> 100	> 100
**5**	43.23 ± 2.03^c^	45.80 ± 2.49^b^
**6**	36.58 ± 0.76^d^	25.96 ± 1.12^d^
Trolox	41.12 ± 1.81^c^	29.09 ± 0.94^c^

IC_50_ values are presented as means ± standard deviation (SD, *n* = 3). Values with the different superscript letters in the same test are significantly different (*p* < 0.05) according to one‐way ANOVA and Duncan's multiple comparison tests. Trolox is used as the positive control.

## 3 Conclusions

In summary, a phytochemical study of VMML yielded five previously undescribed iridoids. Their structures were elucidated through comprehensive spectroscopic analyses, while the absolute configurations were assigned by comparing experimental and calculated ECD spectra. Evaluation of the antioxidant activity of all isolates revealed that compounds **2**, **5**, and **6** exhibited DPPH (IC_50_ = 36.58–53.90 µM) and ABTS (IC_50_ = 25.96–45.80 µM) free radical scavenging activity. Among them, compound **6** exhibited the strongest antioxidant activity. These findings suggested that iridoids from VMML possessed appreciable in vitro free radical scavenging activity, warranting further investigation in cell‐based oxidative stress models to evaluate their biological antioxidant efficacy.

## 4 Experimental

### 4.1 General Experimental Procedures

HRESIMS data were collected on a Bruker MaXis high‐resolution time‐of‐flight system (Bruker, Germany). UV absorption spectra were recorded on a Varian Cary 5000 spectrophotometer, and IR spectra were obtained on a Varian 610/670 FTIR spectrometer (Varian, USA). A Bruker AVANCE‐600 spectrometer (Bruker, Germany) was employed to obtain NMR spectra. Column chromatography (CC) was conducted using ODS (YMC, Japan) and Sephadex LH‐20 (GE Healthcare Biosciences AB, Sweden) as stationary phases.

### 4.2 Plant Material

The fresh leaves of VMML were collected in Yangzhou, Jiangsu Province, China, in May 2024. Authentication of the specimen was performed by Professor M. X. Liu from Yangzhou University. The verified sample (No. YZ‐20240501) was deposited in the authors' laboratory at Yangzhou University, China.

### 4.3 Extraction and Isolation

Extraction of dried VMML (4.2 kg) was achieved by triple methanol maceration (32 L, 24 h each) at room temperature. Concentration of the combined filtrates under vacuum provided 183 g of crude extract. Further purification via silica gel CC, utilizing a CH_2_Cl_2_–MeOH gradient (50:1→1:1, *v/v*), separated the residue into eight fractions (Fr.1–Fr.8). Repeated chromatographic separation of Fr.1 and Fr.3 over silica gel, Sephadex LH‐20, ODS, and preparative HPLC yielded compounds **1** (2 mg), **2** (6 mg), **3** (5 mg), **4** (7 mg), **5** (5 mg), and **6** (8 mg). Detailed isolation procedures are provided in the Supporting Information (Scheme S1).

Viburetin D (**1**): ${\left[\alpha \right]}_{D}^{23}$ = +13.4 (*c* 0.05, MeOH); HRESIMS *m/z* 235.0939 [M+Na]^+^ (calcd for C_11_H_16_O_4_Na^+^, 235.0941) (Figure S1); UV (MeOH) *λ*_max_ nm (log *ε*) 195 (3.02); IR (KBr) *v*_max_ 3326, 2941, 1668, 1385, 1332, 1192, 1125, 1070, 952, 846, and 798 cm^−1^ (Figure S2); 1D/2D NMR data, Figures S3–S9.

Viburetin E (**2**): ${\left[\alpha \right]}_{D}^{23}$ = +41.5 (*c* 0.20, MeOH); HRESIMS *m/z* 251.0886 [M+Na]^+^ (calcd for C_11_H_16_O_5_Na^+^, 251.0890) (Figure S10); UV (MeOH) *λ*_max_ nm (log *ε*) 195 (3.81); IR (KBr) *v*_max_ 3397, 2934, 1626, 1384, 1314, 1194, 1127, 1074, 1011, 950, 851, and 780 cm^−1^ (Figure S11); 1D/2D NMR data, Figures S12–S18.

Viburetin F (**3**): ${\left[\alpha \right]}_{D}^{23}$ = +25.9 (*c* 0.10, MeOH); HRESIMS *m/z* 335.1112 [M+Na]^+^ (calcd for C_15_H_20_O_7_Na^+^,335.1101) (Figure S19); UV (MeOH) *λ*_max_ nm (log *ε*) 192 (3.87); IR (KBr) *v*_max_ 3464, 2959, 1743, 1373, 1247, 1129, 1068, 1024, 954, 855, 785 cm^−1^ (Figure S20); 1D/2D NMR data, Figures S21–S27.

Viburetin G (**4**): ${\left[\alpha \right]}_{D}^{23}$ = −30.1 (*c* 0.15, MeOH); HRESIMS *m/z* 335.1108 [M+Na]^+^ (calcd for C_15_H_20_O_7_Na^+^, 335.1101) (Figure S28); UV (MeOH) *λ*_max_ nm (log *ε*) 196 (3.26); IR (KBr) *v*_max_ 3450, 2939, 1742, 1391, 1246, 1132, 1079, 1034, 968, 867, and 791 cm^−1^ (Figure S29); 1D/2D NMR data, Figures S30–S36.

Viburetin H (**5**): ${\left[\alpha \right]}_{D}^{23}$ = +22.8 (*c* 0.10, MeOH); HRESIMS *m/z* 209.1148 [M+Na]^+^ (calcd for C_10_H_18_O_3_Na^+^, 209.1148) (Figure S37); UV (MeOH) *λ*_max_ nm (log *ε*) 193 (2.53); IR (KBr) *v*_max_ 3338, 2944, 1650, 1448, 1024, 900, and 649 cm^−1^ (Figure S38); 1D/2D NMR data, Figures S39–S45.

### 4.4 ECD Calculations

The initial conformations of compounds **1**–**5** were generated using MOE software via a systematic conformational search, based on the structures deduced from NMR data and built with Chem3D. All feasible conformers were optimized at the B3LYP/6‐31G (d,p) level of density functional theory (DFT) using Gaussian 16. Subsequent time‐dependent DFT (TDDFT) calculations were performed at the CAM‐B3LYP/6‐311G (d,p) level with the CPCM solvent model in MeOH. The calculated ECD spectra of individual conformers were Boltzmann‐weighted according to their relative free energies and simulated in SpecDis 1.71 with a UV correction applied as needed to align the simulated spectra with the experimental data.

### 4.5 Antioxidant Activity Assays

The DPPH• and ABTS•^+^ radical‐scavenging activities of compounds **1**–**6** were determined colorimetrically (*λ* = 517 and 734 nm, respectively) following established procedures [20, 21], with Trolox as the reference and data expressed as IC_50_ values. The experimental details were provided in the Supporting Information.

### 4.6 Statistical Analysis

The data from biological assays were presented as mean ± standard deviation (SD, *n* = 3), which were analyzed by one‐way ANOVA and Duncan's multiple tests in SPSS 25.0 software, with *p* < 0.05 considered statistically significant.

## Author Contributions

**Ya‐Jiao Chen**: investigation, methodology, writing – original draft. **Hong‐Juan Zhou**: formal analysis, validation. **Jia Chen**: methodology, software, validation. **Jian‐Hua Shao**: funding acquisition, supervision. **Chun‐Chao Zhao**: project administration, funding acquisition, writing – review and editing.

## Conflicts of Interest

The authors declare no conflicts of interest.

## Supporting information




**Supporting File 1**: cbdv71649‐sup‐0001‐SuppMat.pdf

## Data Availability

The data that support the findings of this study are available in the Supporting Information of this article.
