# Supplementary material for: Five New Iridoids From the Leaves of Viburnum macrocephalum f. macrocephalum and Their Free Radical Scavenging Activity
Source: Chem Biodivers. 2026 Sep 3;23(9):e71649. doi: 10.1002/cbdv.71649 (PMC13539465; doi:10.1002/cbdv.71649)
Supplement: Supplementary file 1 [file CBDV-23-e71649-s001.pdf]

SUPPLEMENTARY MATERIAL

Five New Iridoids from the Leaves of *Viburnum macrocephalum* f. *macrocephalum* and their Free Radical Scavenging Activity

Ya-Jiao Chen[#], Hong-Juan Zhou[#], Jia Chen, Jian-Hua Shao*, and Chun-Chao Zhao*

College of Bioscience and Biotechnology, Yangzhou University, Yangzhou 225009, China

*Corresponding Authors. **Jian-Hua Shao**: College of Bioscience and Biotechnology, Yangzhou University, Yangzhou 225009, China; e-mail: jhshao@yzu.edu.cn; phone: +86-514-87979054. **Chun-Chao Zhao**: College of Bioscience and Biotechnology, Yangzhou University, Yangzhou 225009, China; e-mail: cczhao@yzu.edu.cn; phone: +86-514-87991556.

List of supporting information

**1.**	**Figure S1**. The (+)-HRESIMS Spectrum of Compound **1**
**2.**	**Figure S2**. The IR Spectrum of Compound **1**
**3.**	**Figure S3**. The ^{1}H NMR Spectrum of Compound **1** in CD_3OD
**4.**	**Figure S4**. The ^{13}C NMR Spectrum of Compound **1** in CD_3OD
**5.**	**Figure S5**. The DEPT 135 Spectrum of Compound **1** in CD_3OD
**6.**	**Figure S6**. The HSQC Spectrum of Compound **1** in CD_3OD
**7.**	**Figure S7**. The HMBC Spectrum of Compound **1** in CD_3OD
**8.**	**Figure S8**. The ^{1}H-^{1}H COSY Spectrum of Compound **1** in CD_3OD
**9.**	**Figure S9**. The NOESY Spectrum of Compound **1** in CD_3OD
**10.**	**Figure S10**. The (+)-HRESIMS Spectrum of Compound **2**
**11.**	**Figure S11**. The IR Spectrum of Compound **2**
**12.**	**Figure S12**. The ^{1}H NMR Spectrum of Compound **2** in $CDCl_3$
**13.**	**Figure S13**. The ^{13}C NMR Spectrum of Compound **2** in $CDCl_3$
**14.**	**Figure S14**. The DEPT 135 Spectrum of Compound **2** in $CDCl_3$
**15.**	**Figure S15**. The HSQC Spectrum of Compound **2** in $CDCl_3$
**16.**	**Figure S16**. The HMBC Spectrum of Compound **2** in $CDCl_3$
**17.**	**Figure S17**. The ^{1}H-^{1}H COSY Spectrum of Compound **2** in $CDCl_3$
**18.**	**Figure S18**. The NOESY Spectrum of Compound **2** in $CDCl_3$
**19.**	**Figure S19**. The (+)-HRESIMS Spectrum of Compound **3**
**20.**	**Figure S20**. The IR Spectrum of Compound **3**
**21.**	**Figure S21**. The ^{1}H NMR Spectrum of Compound **3** in CD_3OD
**22.**	**Figure S22**. The ^{13}C NMR Spectrum of Compound **3** in CD_3OD
**23.**	**Figure S23**. The DEPT 135 Spectrum of Compound **3** in CD_3OD
**24.**	**Figure S24**. The HSQC Spectrum of Compound **3** in CD_3OD
**25.**	**Figure S25**. The HMBC Spectrum of Compound **3** in CD_3OD
**26.**	**Figure S26**. The ^{1}H-^{1}H COSY Spectrum of Compound **3** in CD_3OD

**27.**	**Figure S27**. The NOESY Spectrum of Compound **3** in CD_3OD
**28.**	**Figure S28**. The (+)-HRESIMS Spectrum of Compound **4**
**29.**	**Figure S29**. The IR Spectrum of Compound **4**
**30.**	**Figure S30**. The ^1H NMR Spectrum of Compound **4** in CD_3OD
**31.**	**Figure S31**. The ^{13}C NMR Spectrum of Compound **4** in CD_3OD
**32.**	**Figure S32**. The DEPT 135 Spectrum of Compound **4** in CD_3OD
**33.**	**Figure S33**. The HSQC Spectrum of Compound **4** in CD_3OD
**34.**	**Figure S34**. The HMBC Spectrum of Compound **4** in CD_3OD
**35.**	**Figure S35**. The ^1H-^1H COSY Spectrum of Compound **4** in CD_3OD
**36.**	**Figure S36**. The NOESY Spectrum of Compound **4** in CD_3OD
**37.**	**Figure S37**. The (+)-HRESIMS Spectrum of Compound **5**
**38.**	**Figure S38**. The IR Spectrum of Compound **5**
**39.**	**Figure S39**. The ^1H NMR Spectrum of Compound **5** in DMSO-d_6
**40.**	**Figure S40**. The ^{13}C NMR Spectrum of Compound **5** in DMSO-d_6
**41.**	**Figure S41**. The DEPT 135 Spectrum of Compound **5** in DMSO-d_6
**42.**	**Figure S42**. The HSQC Spectrum of Compound **5** in DMSO-d_6
**43.**	**Figure S43.** The HMBC Spectrum of Compound **5** in DMSO-d_6
**44.**	**Figure S44**. The ^1H-^1H COSY Spectrum of Compound **5** in DMSO-d_6
**45.**	**Figure S45**. The NOESY Spectrum of Compound **5** in DMSO-d_6

S1. Extraction and Isolation

Extraction of dried VMML (4.2 kg) was achieved by triple methanol maceration (32 L, 24 h each) at room temperature. Concentration of the combined filtrates under vacuum provided 183 g of crude extract. Further purification via silica gel CC, utilizing a CH_2Cl_2–MeOH gradient (50:1→1:1, v/v), separated the residue into eight fractions (Fr.1–Fr.8). Fraction 1 was fractionated by silica gel CC, eluted with a gradient of PE–EtOAc (40:1→1:1, v/v), to yield four subfractions (Fr.1-1–Fr.1-4). Fr.1-1 was subjected to Sephadex LH-20 CC with CH_2Cl_2–MeOH (2:1, v/v) to obtain three subfractions (Fr.1-1-1–Fr.1-1-3). Fr.1-2 was purified by Sephadex LH-20 CC (CH_2Cl_2–MeOH, 2:1, v/v), followed by ODS CC with a MeOH–H_2O gradient (40:60→90:10, v/v), and finally by preparative HPLC (MeOH–H_2O, 41:59, v/v; 9.9 mL/min) to yield compounds **3** (5 mg, t_R= 44.7 min) and **4** (7 mg, t_R = 46.1 min). Fr.1-3 was chromatographed on Sephadex LH-20 with CH_2Cl_2–MeOH (2:1, v/v) to give compound **1** (2 mg). Fr.1-4 was separated on a Sephadex LH-20 column using CH_2Cl_2–MeOH (2:1, v/v) and further purified by ODS CC with a MeOH–H_2O gradient (45:55→90:10, v/v) to obtain compounds **2** (6 mg) and **6** (8 mg). Fraction 3 was subjected to silica gel CC (PE–EtOAc, 30:1→1:1, v/v) to give four subfractions (Fr.3-1–Fr.3-4); Fr.3-1 was further purified on Sephadex LH-20 (CH_2Cl_2–MeOH, 2:1, v/v) and then by preparative HPLC (MeOH–H_2O, 31:69, v/v; 9.9 mL/min) to yield compound **5** (5 mg, t_R = 31.4 min).

S2. DPPH Radical Scavenging Assay

The DPPH radical scavenging activity was evaluated based on a previously reported method with slight modifications [1]. Briefly, 100 μL of sample solutions at various concentrations were added to a 96-well microplate, followed by 100 μL of 0.2 mM DPPH solution. The mixture was vortexed and incubated in the dark at 37°C for 30 min, after which absorbance was measured at 517 nm. Trolox served as the positive control, while a DPPH-only solution acted as the negative control; pure solvent was used for instrument zeroing. All assays were performed in triplicate. Results are expressed as the IC_{50} value, defined as the sample concentration required to scavenge 50% of the DPPH radicals.

S3. ABTS Radical Scavenging Assay

ABTS radical scavenging activity was evaluated following a modified protocol [2]. An $ABTS^{\bullet +}$ stock solution was prepared by mixing 7 mM ABTS and 2.45 mM potassium persulfate in water, then incubating in the dark at room temperature for 12–16 h. This stock was diluted with absolute ethanol to prepare the working solution. In a 96-well plate, 100 μL of sample solution was mixed with 100 μL of $ABTS^{\bullet +}$ working solution. The mixture was incubated at 37°C in the dark for 30 min, and absorbance was measured at 734 nm. Trolox served as the positive control, while a reagent-only solution served as the negative control; pure solvent was used for zeroing. Experiments were conducted in triplicate, and results are reported as IC_{50} values.

Reference

[1] M. J. Goswami, R. Chakrabarty, B. Das and D. Kakati, ‘Isolation and Characterisation of Bioactive Steroidal Compounds from the Leaf Extract of *Sarcochlamys pulcherrima*’, *Chemistry & Biodiversity* 22 (2025): e202500100, https://doi.org/10.1002/cbdv.202500100.

[2] X. X. Huang, M. Bai, L. Zhou, et al. ‘Food Byproducts as A New and Cheap Source of Bioactive Compounds: Lignans with Antioxidant and Anti-inflammatory Properties from *Crataegus pinnatifida* Seeds’, *Journal of Agricultural and Food Chemistry* 63 (2015): 7252–7260, https://doi.org/10.1021/acs.jafc.5b02835.

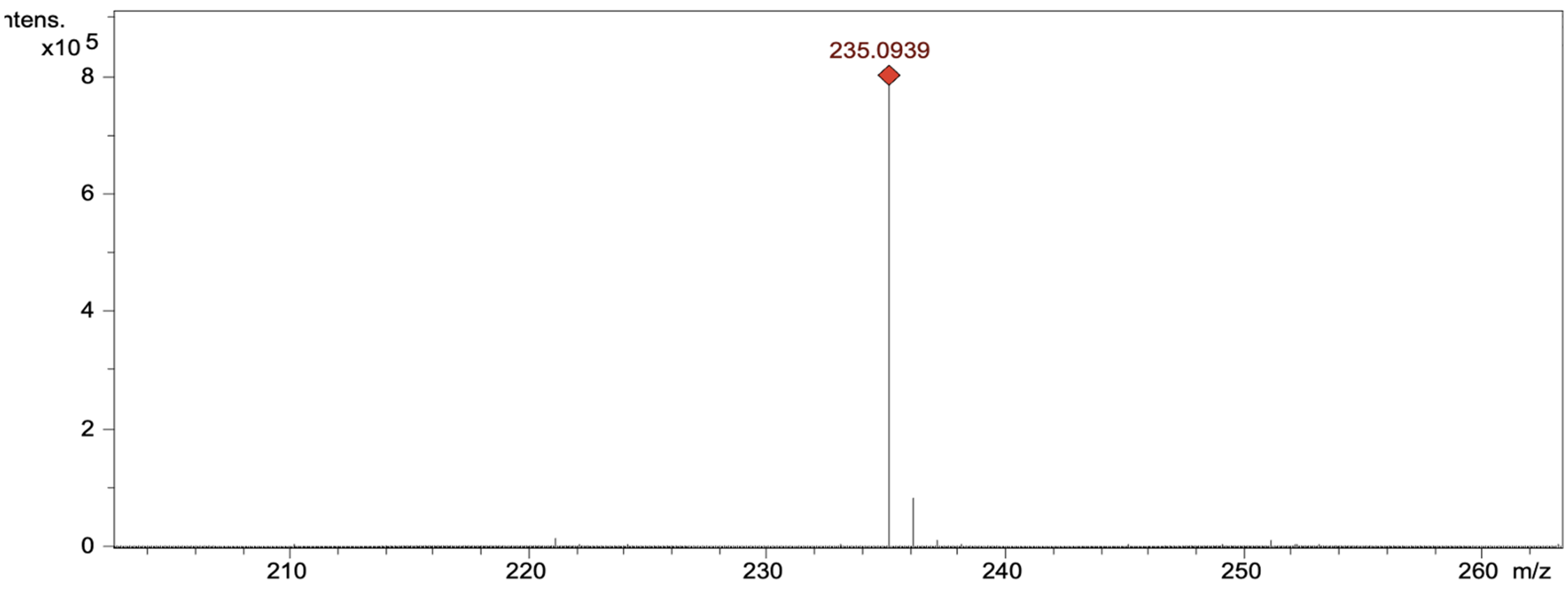

Meas. m/z	#	Formula	Score	m/z	err [mDa]	err [ppm]	mSigma	rdb	e⁻ Conf	N-Rule
235.0939	1	C 11 H 16 Na O 4	100	235.0941	0.2	0.7	7.5	3.5	even	ok

Figure S1. The (+)-HRESIMS Spectrum of Compound **1**

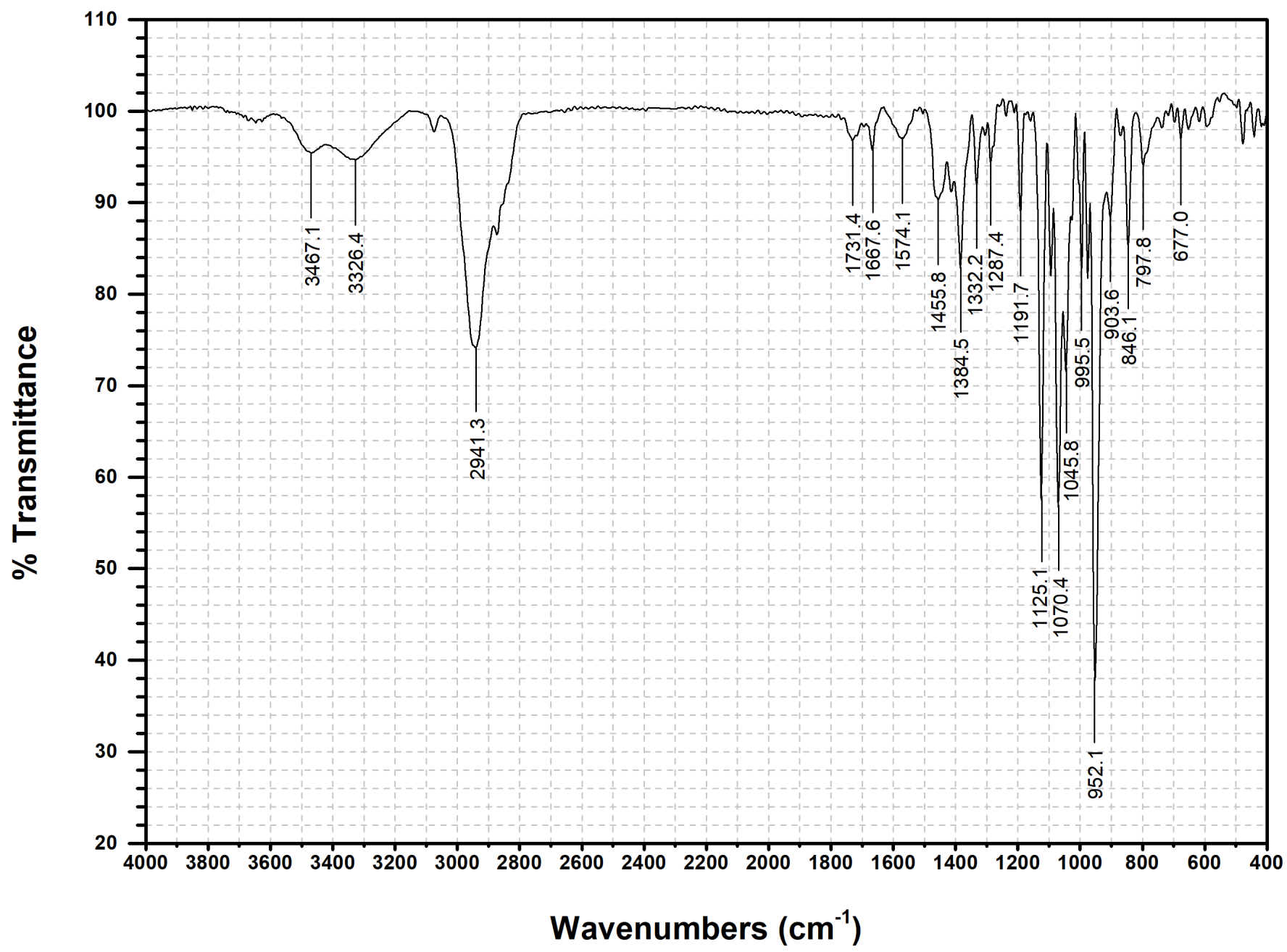

Figure S2. The IR Spectrum of Compound **1**

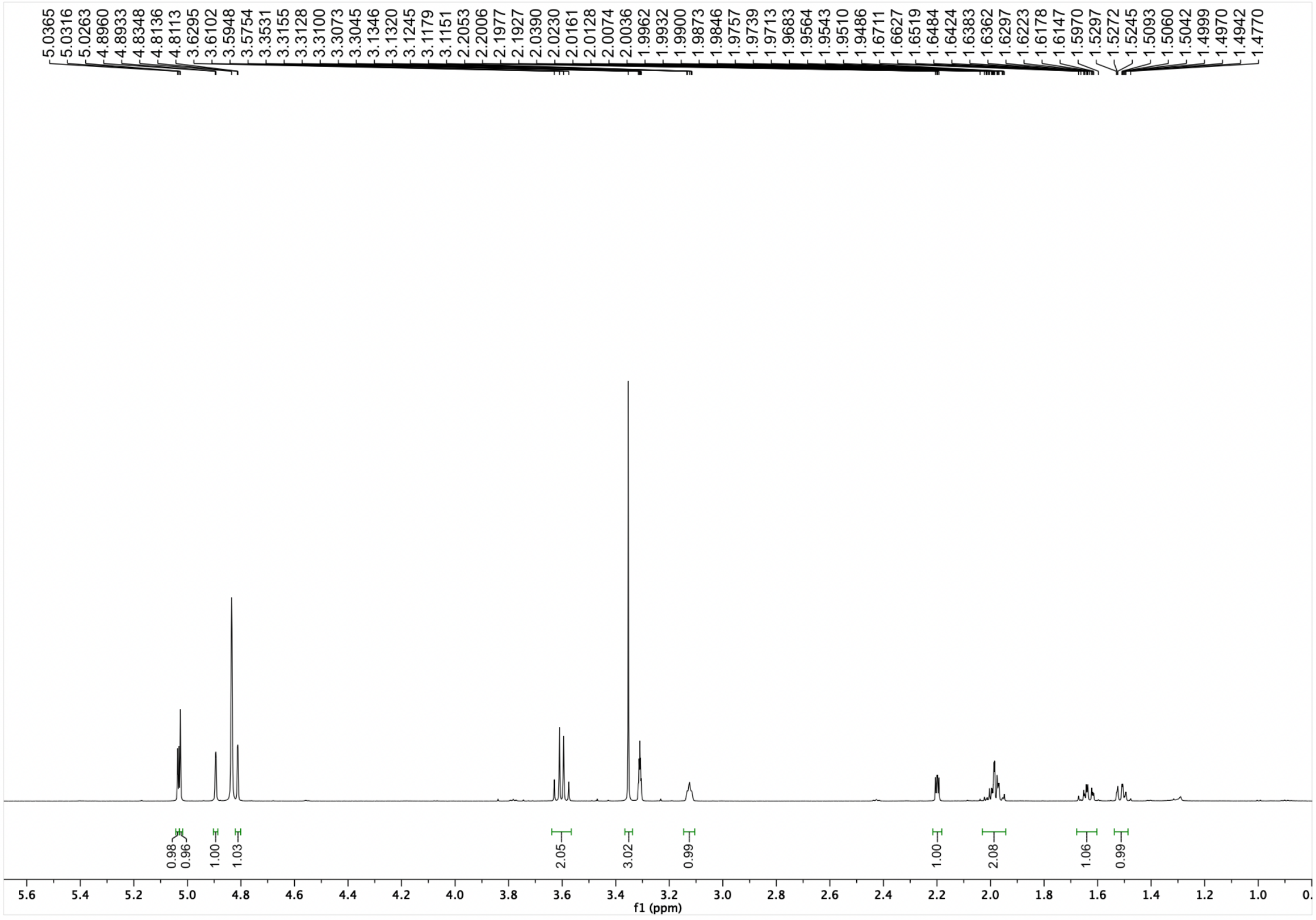

Figure S3. The ^{1}H NMR Spectrum of Compound **1** in CD_3OD

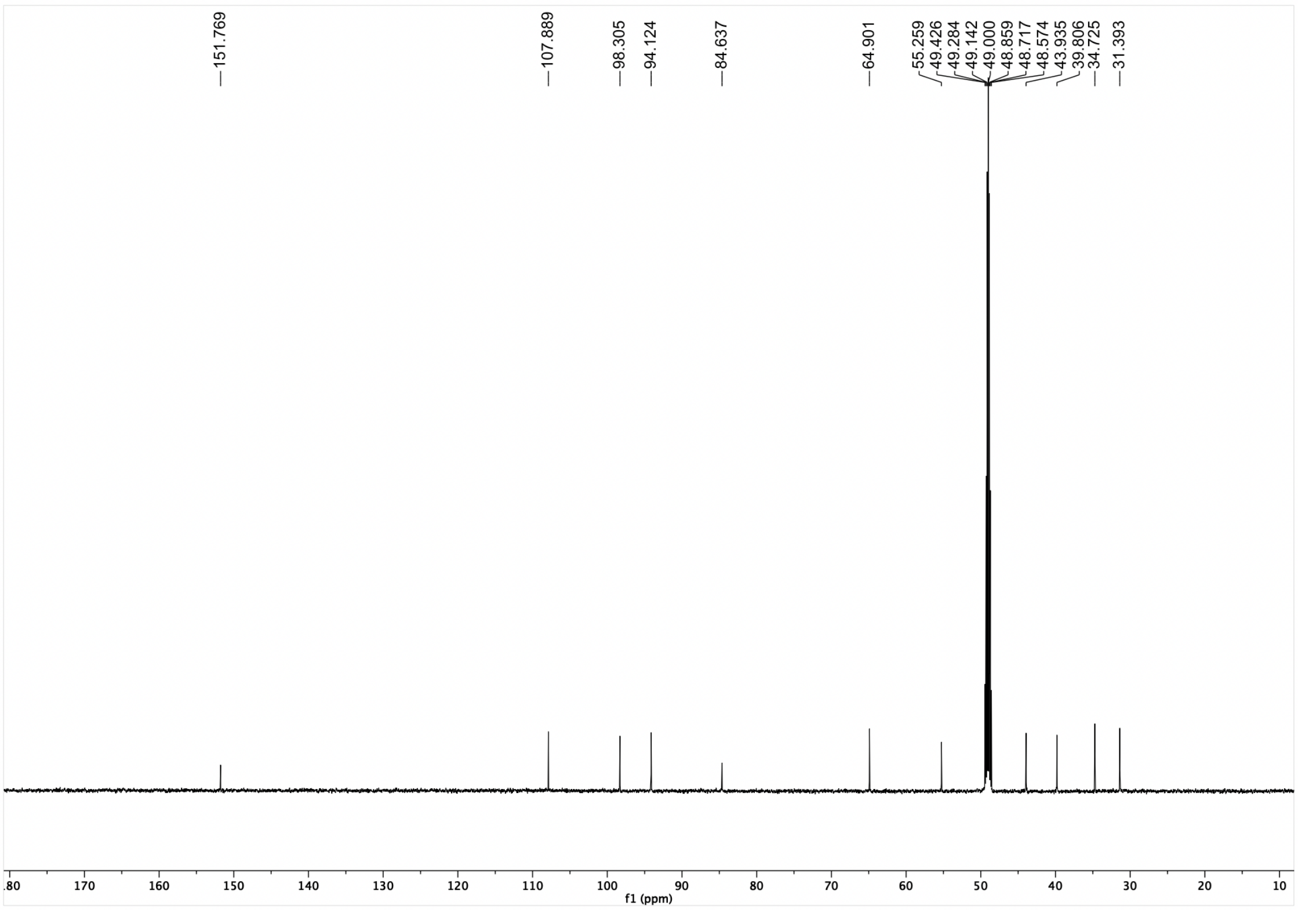

Figure S4. The ^{13}C NMR Spectrum of Compound **1** in CD_3OD

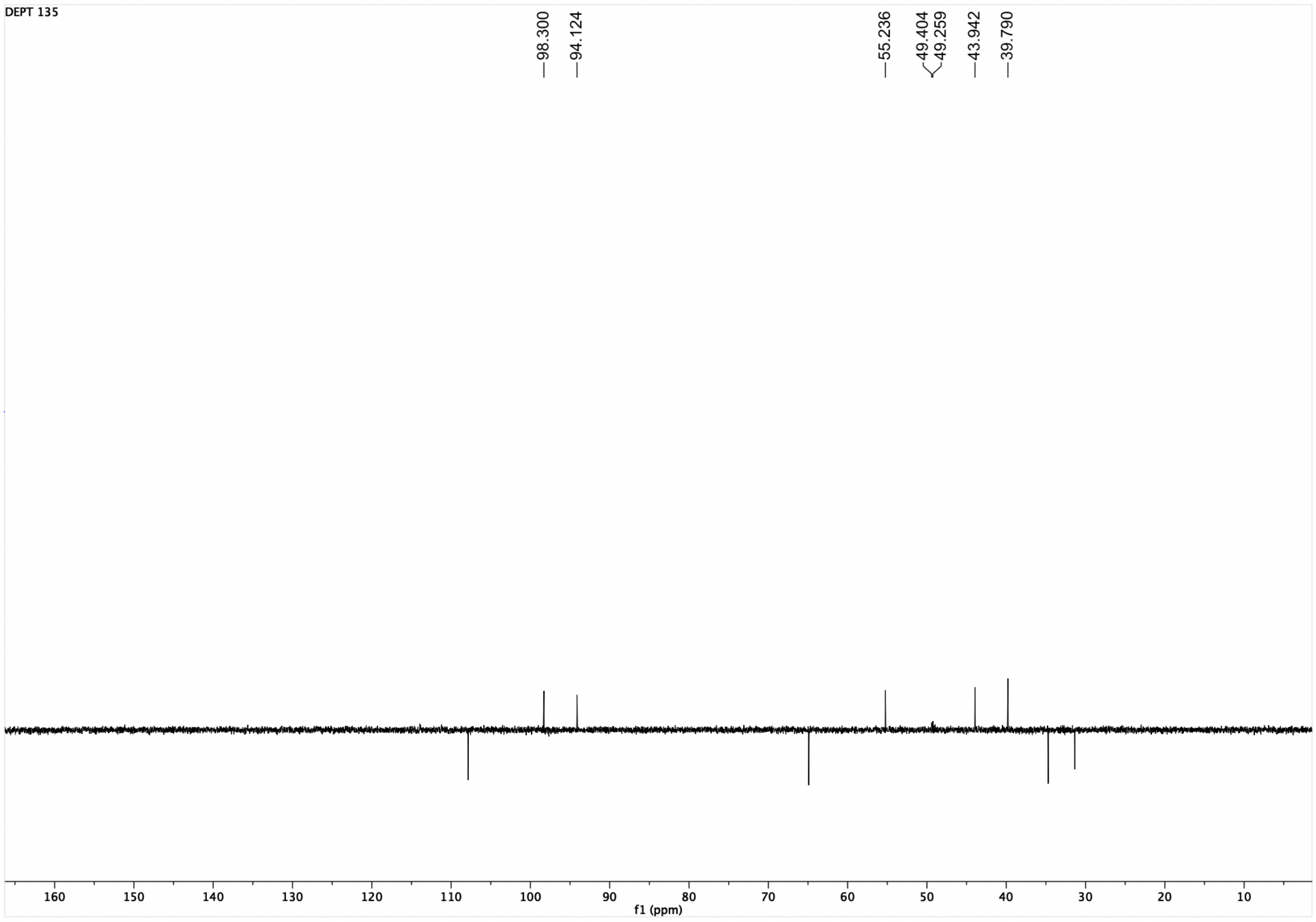

Figure S5. The DEPT 135 Spectrum of Compound **1** in CD_3OD

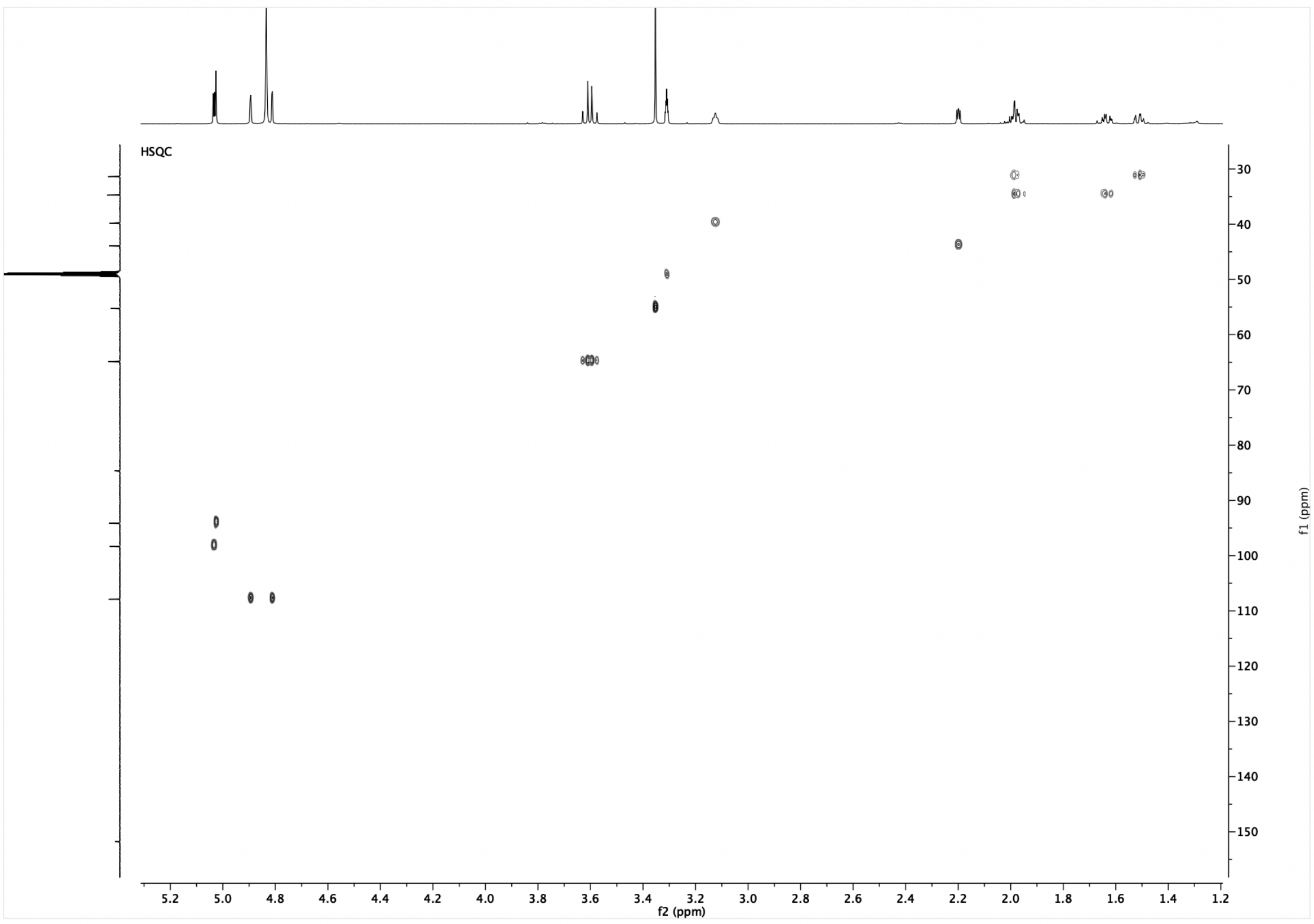

Figure S6. The HSQC Spectrum of Compound **1** in CD_3OD

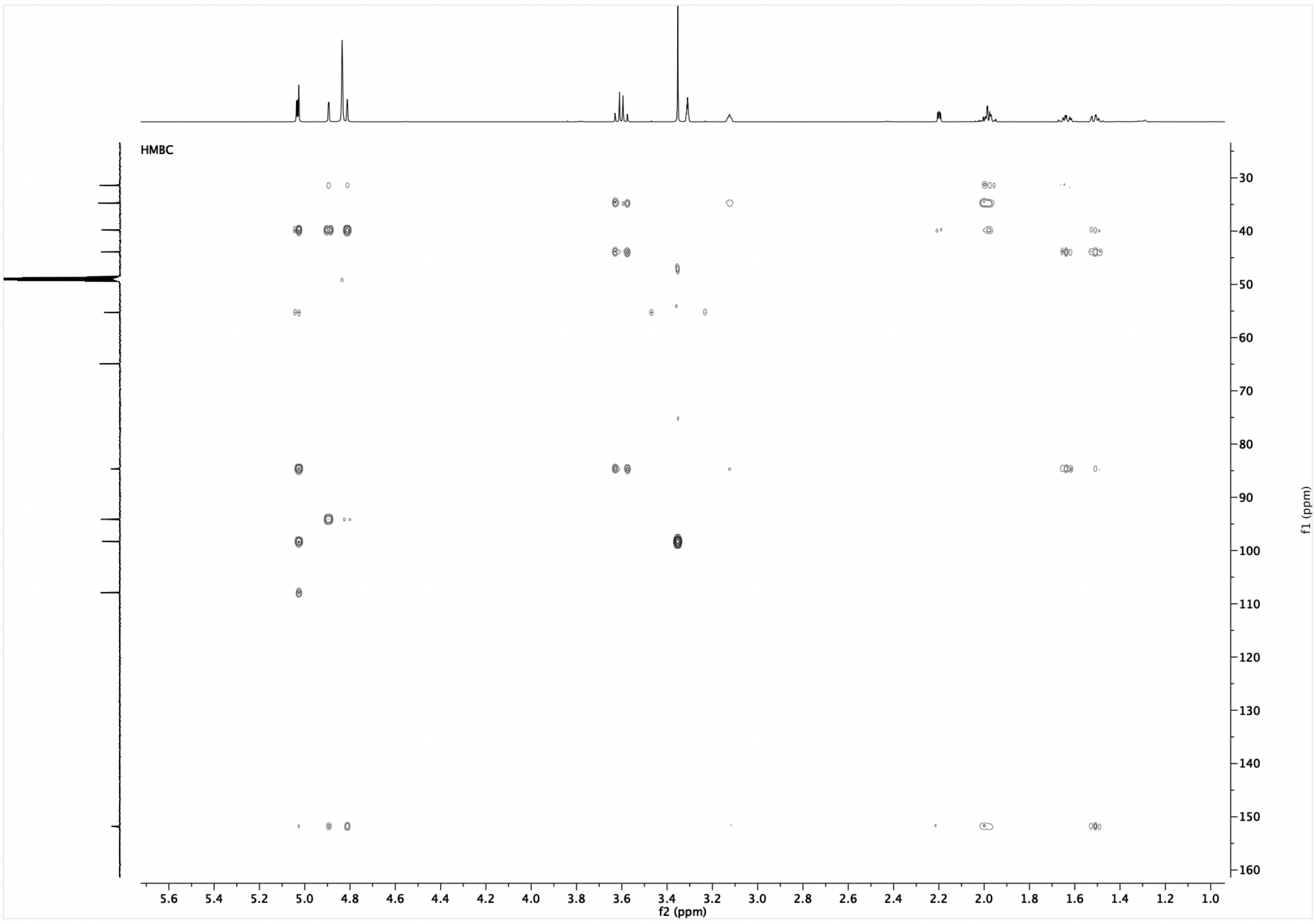

Figure S7. The HMBC Spectrum of Compound **1** in CD_3OD

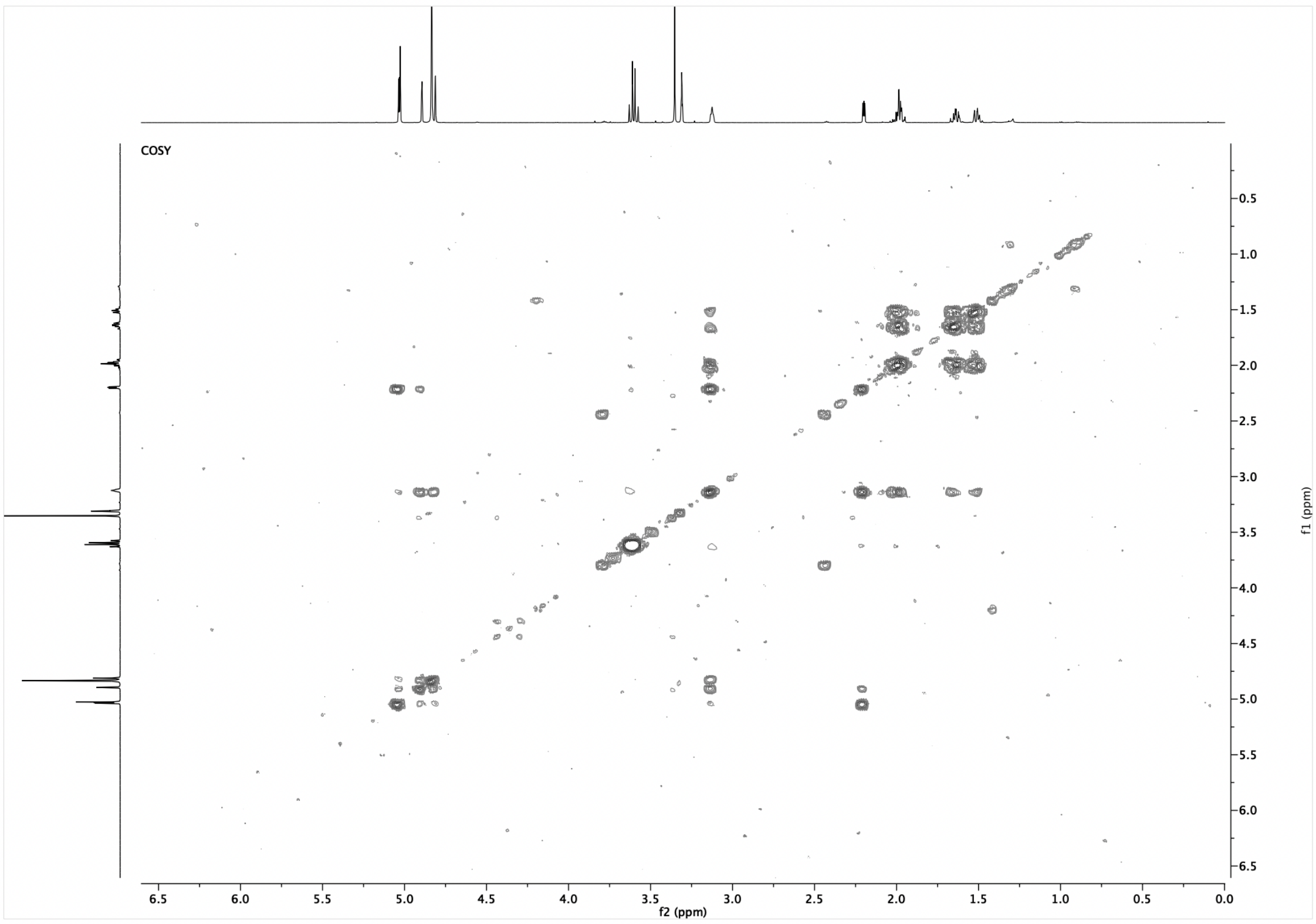

Figure S8. The ^1H-^1H COSY Spectrum of Compound **1** in CD_3OD

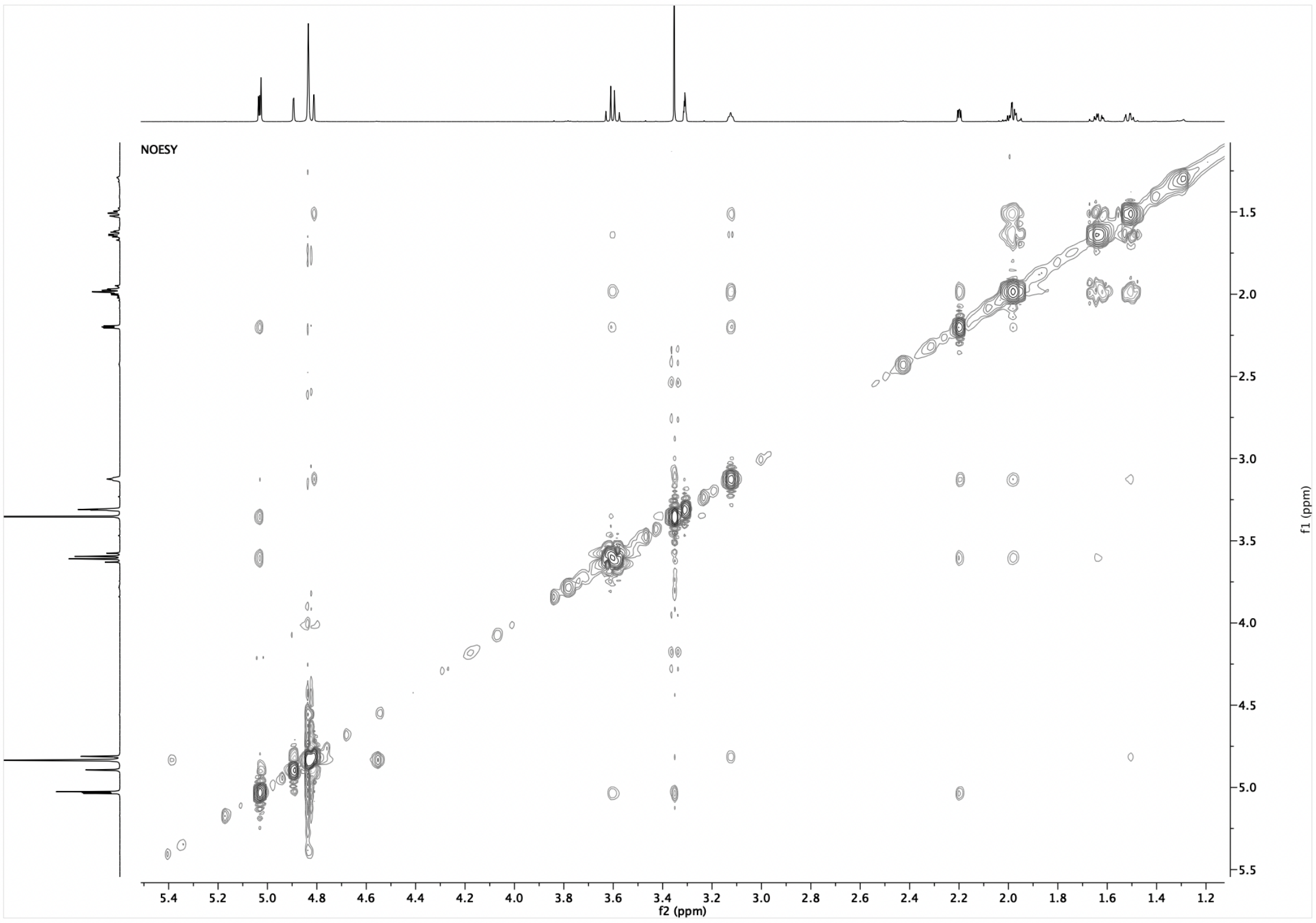

Figure S9. The NOESY Spectrum of Compound **1** in CD_3OD

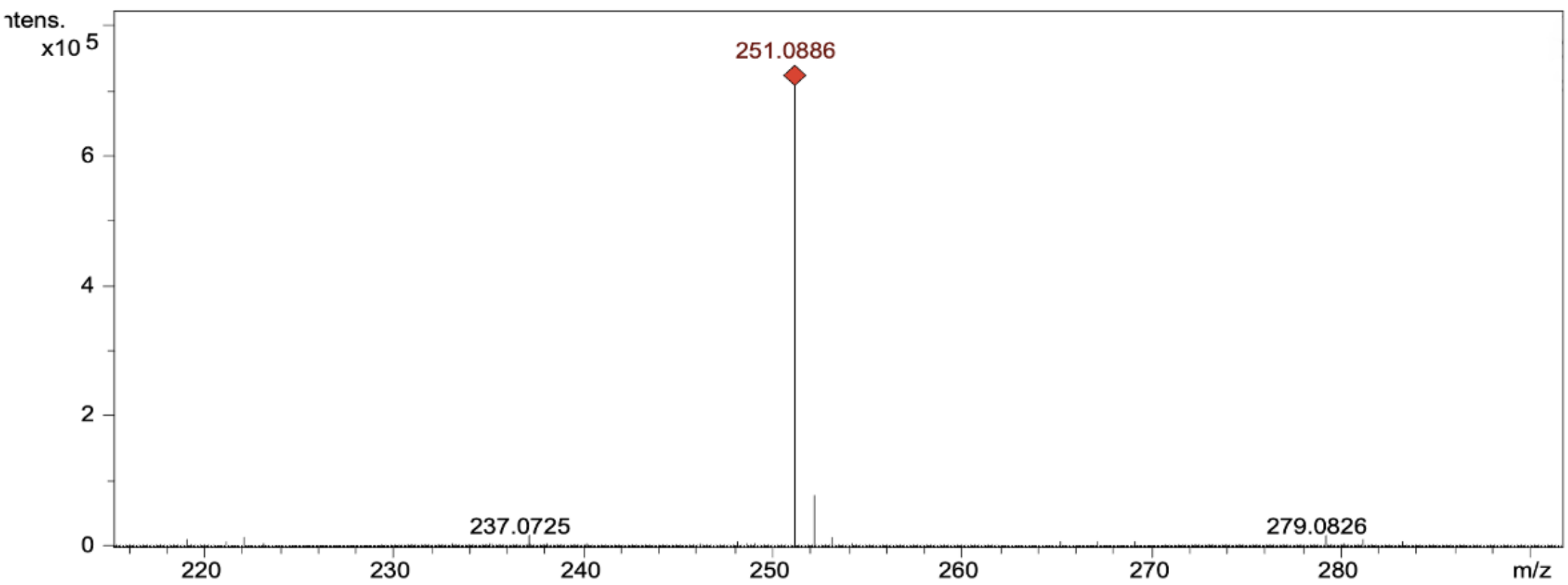

Meas. m/z	#	Formula	Score	m/z	err [mDa]	err [ppm]	mSigma	rdb	e⁻ Conf	N-Rule
251.0886	1	C 11 H 16 Na O 5	100	251.089	0.4	1.6	5.5	3.5	even	ok

Figure S10. The (+)-HRESIMS Spectrum of Compound **2**

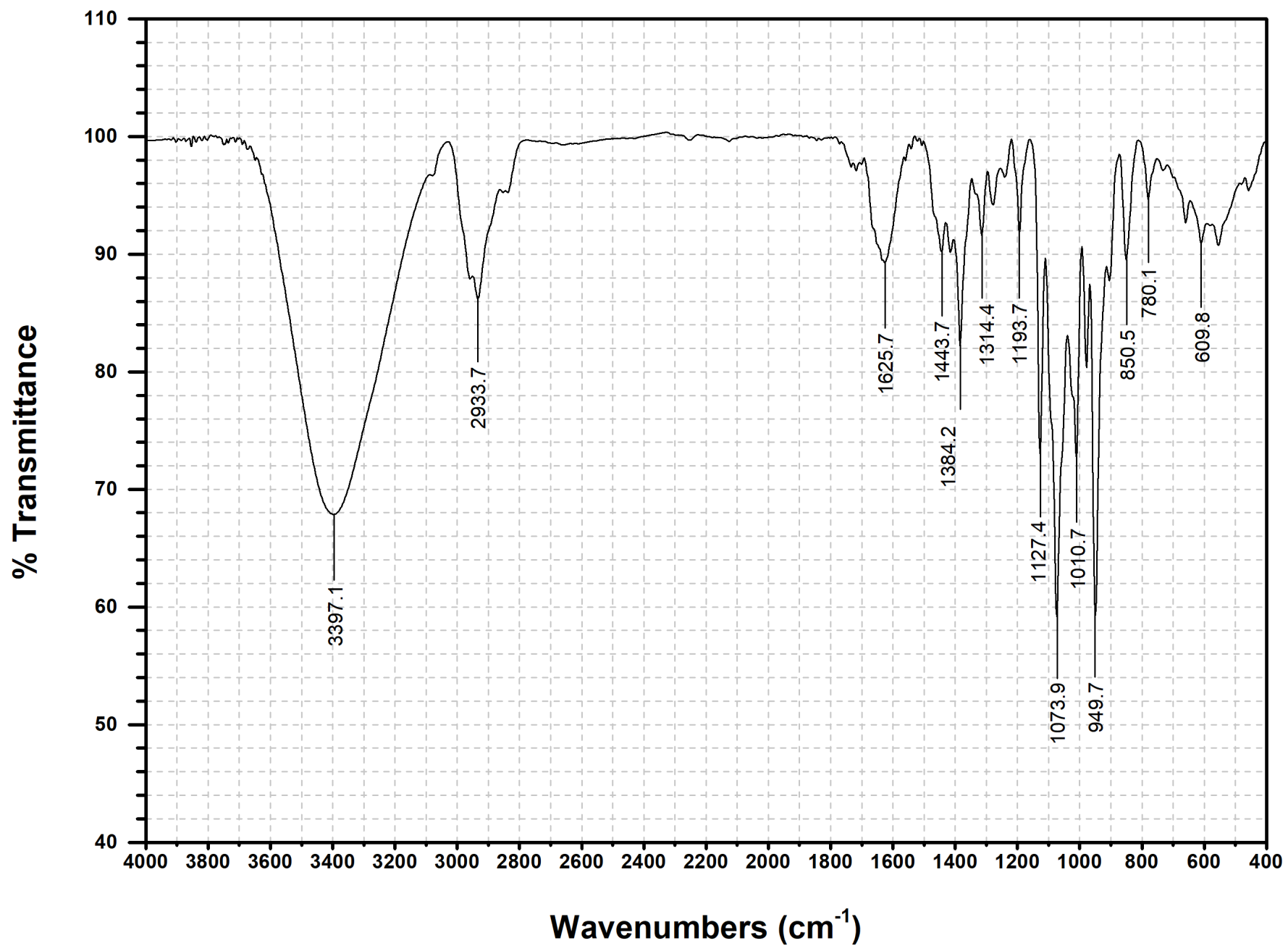

Figure S11. The IR Spectrum of Compound **2**

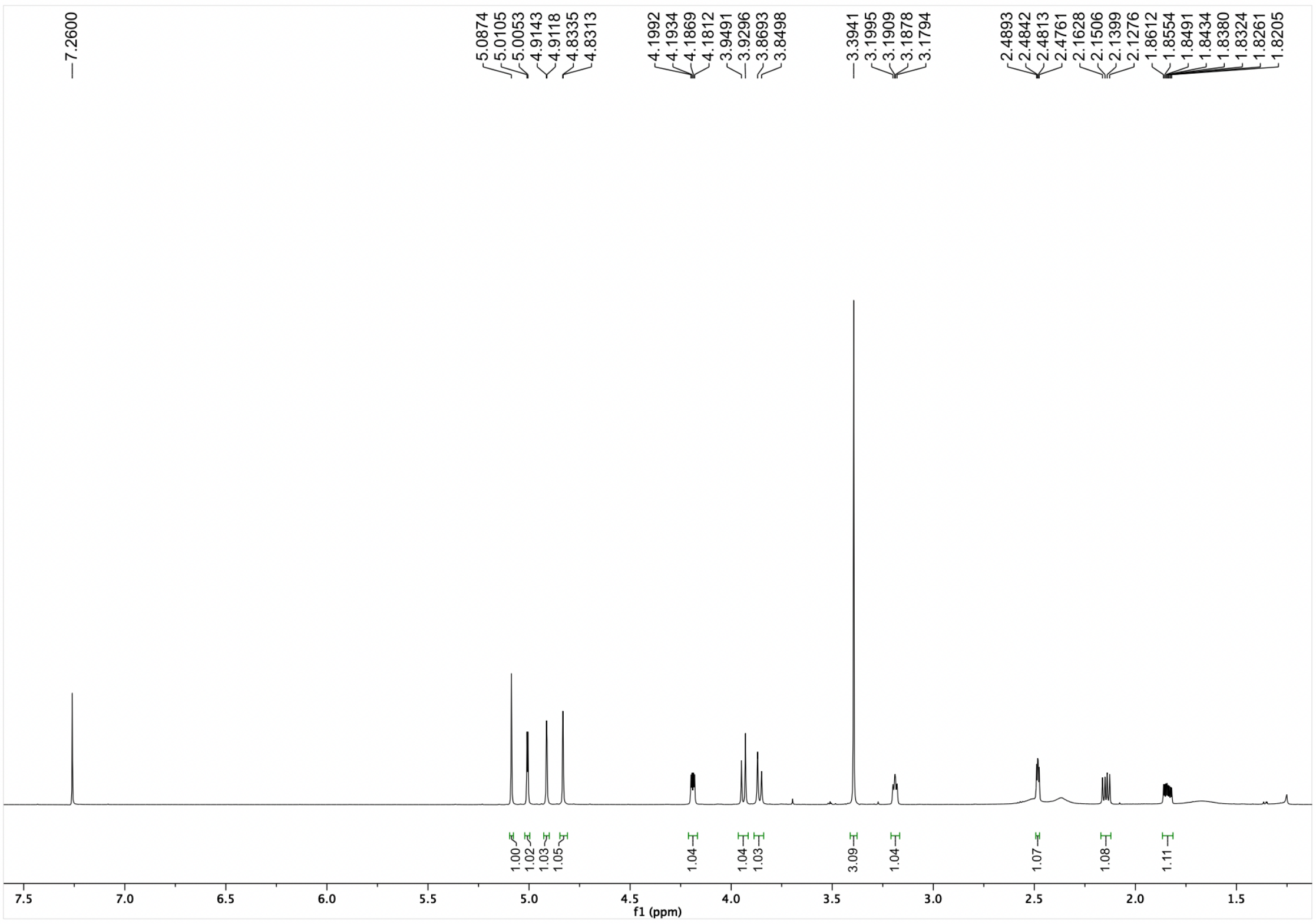

Figure S12. The ^{1}H NMR Spectrum of Compound **2** in $CDCl_3$

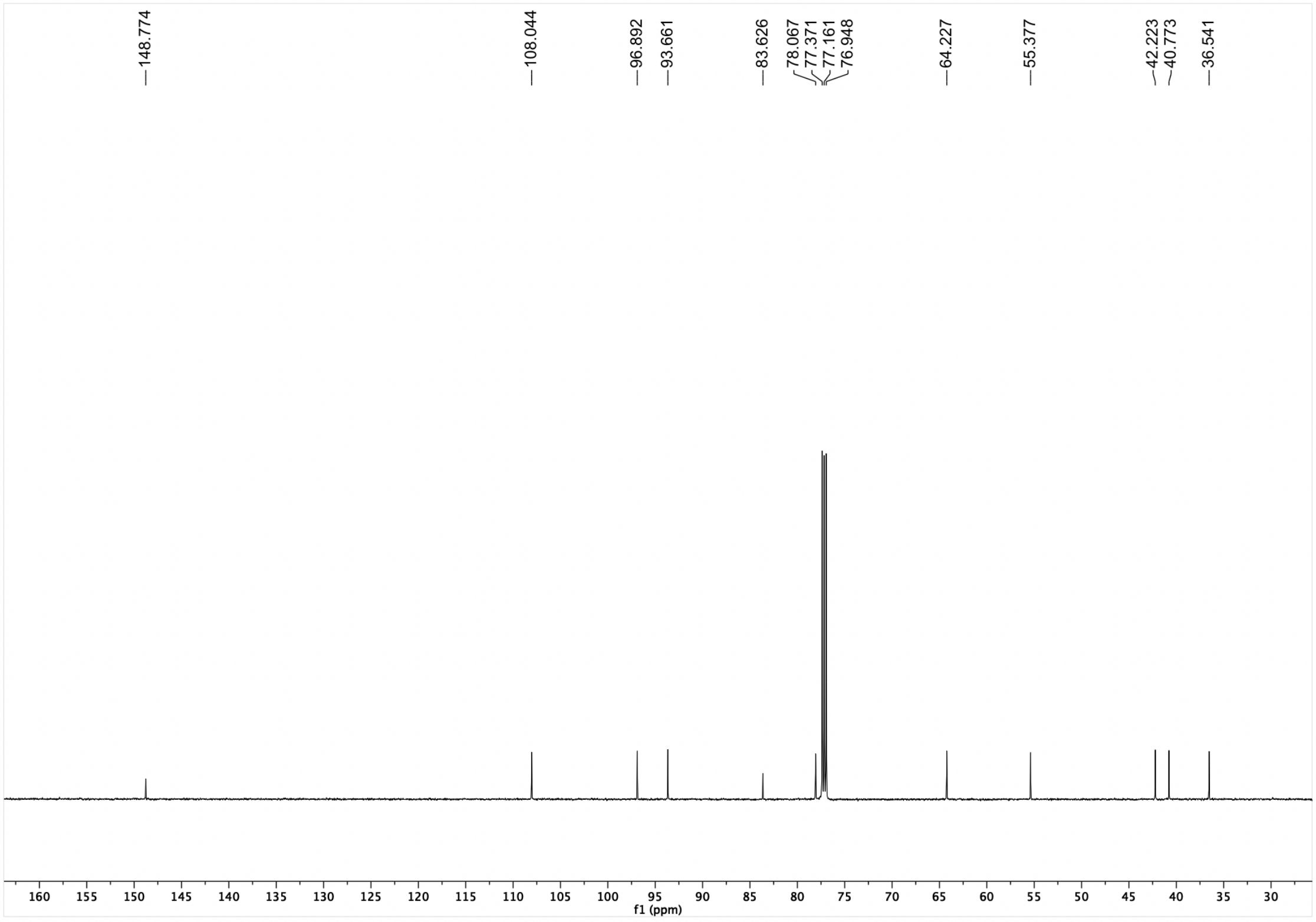

Figure S13. The ^{13}C NMR Spectrum of Compound **2** in $CDCl_3$

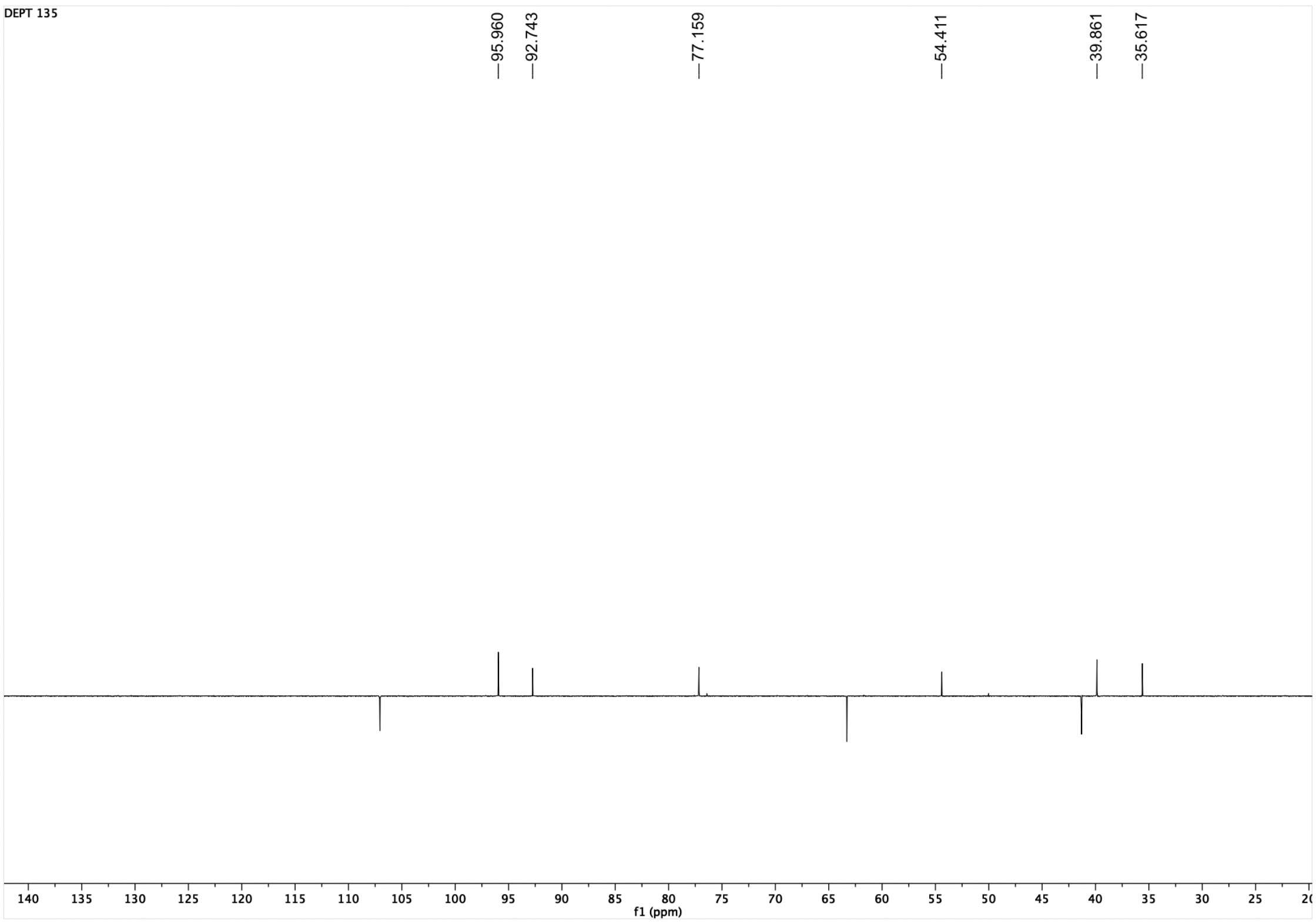

Figure S14. The DEPT 135 Spectrum of Compound **2** in $CDCl_3$

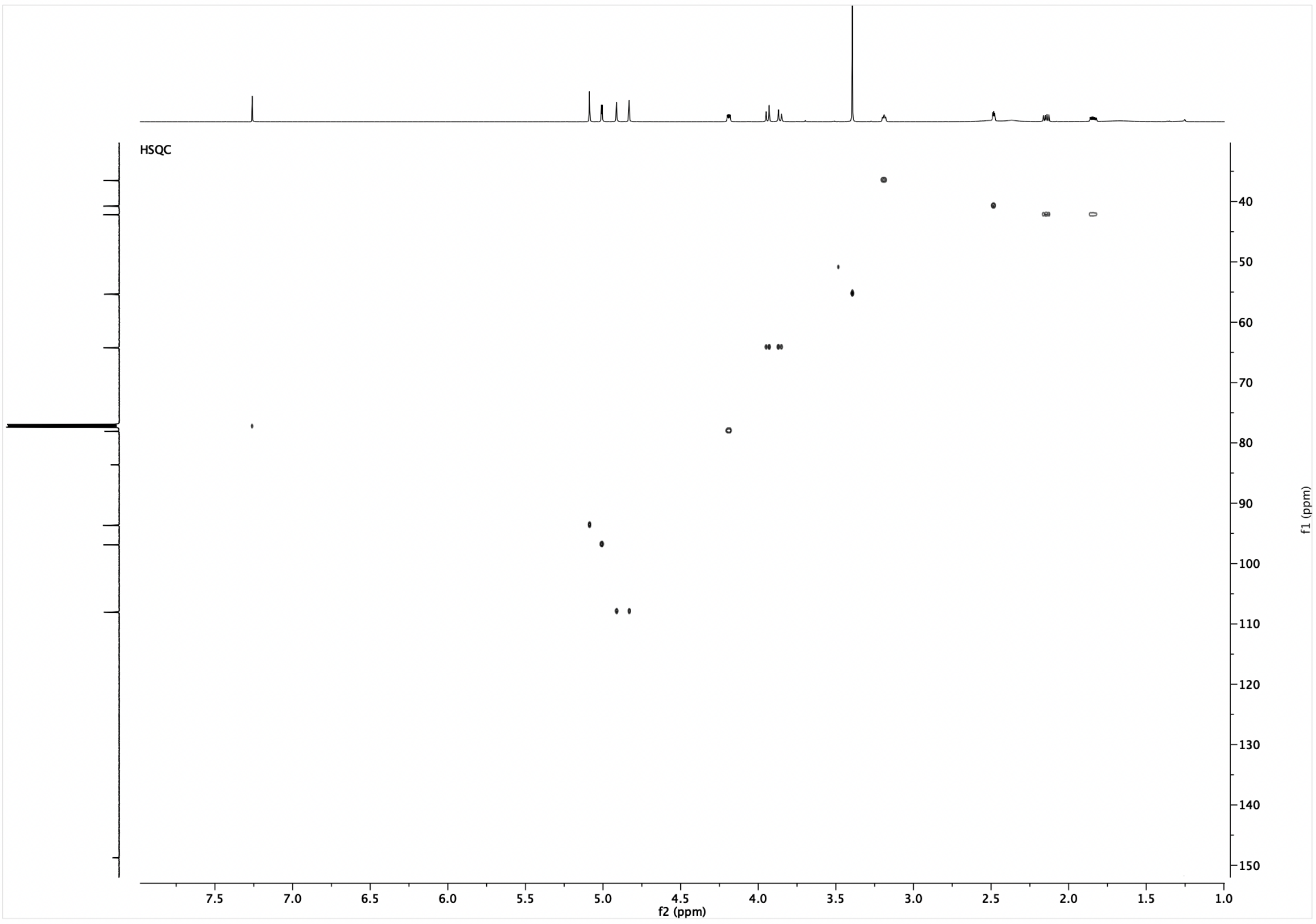

Figure S15. The HSQC Spectrum of Compound **2** in $CDCl_3$

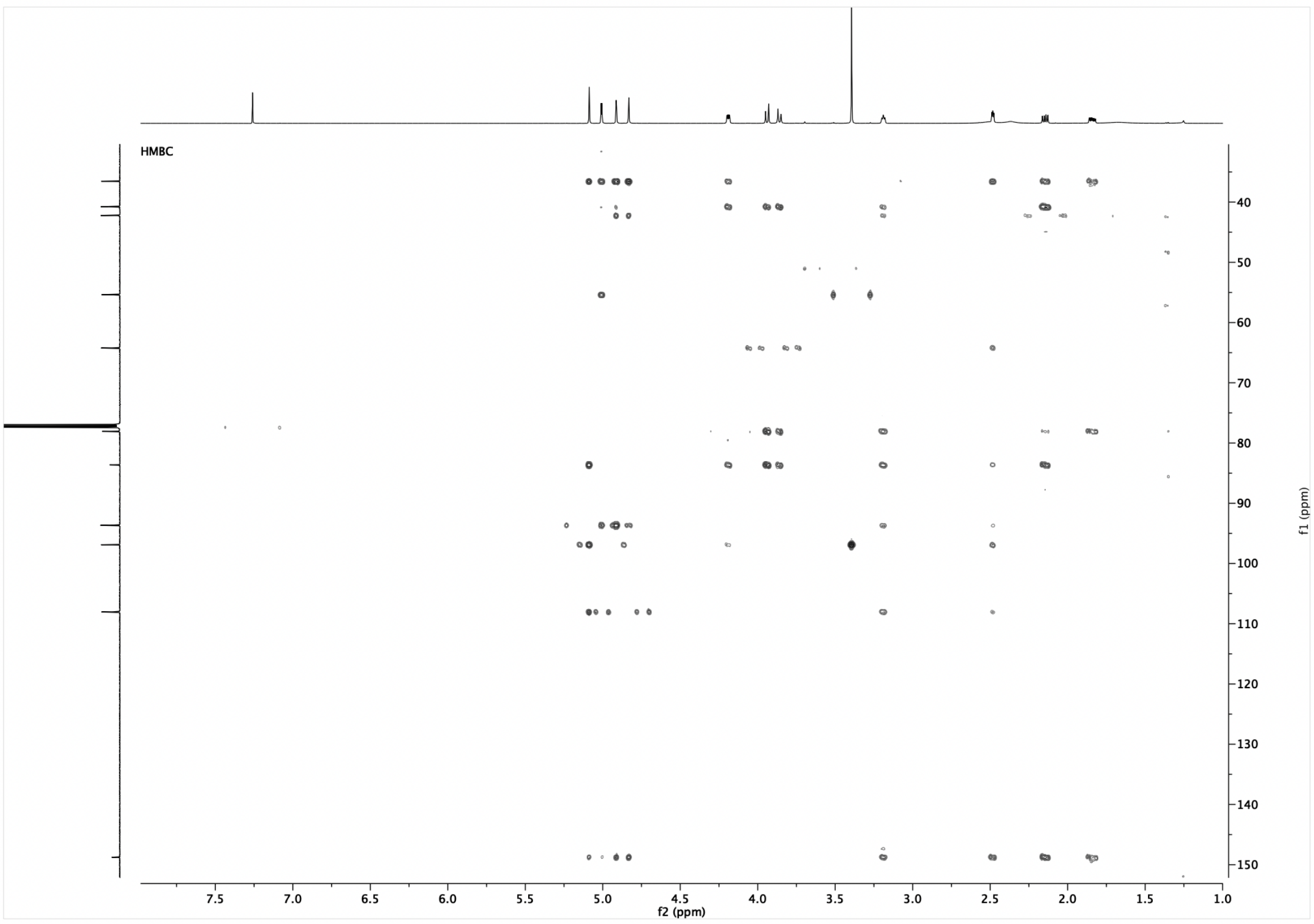

Figure S16. The HMBC Spectrum of Compound **2** in $CDCl_3$

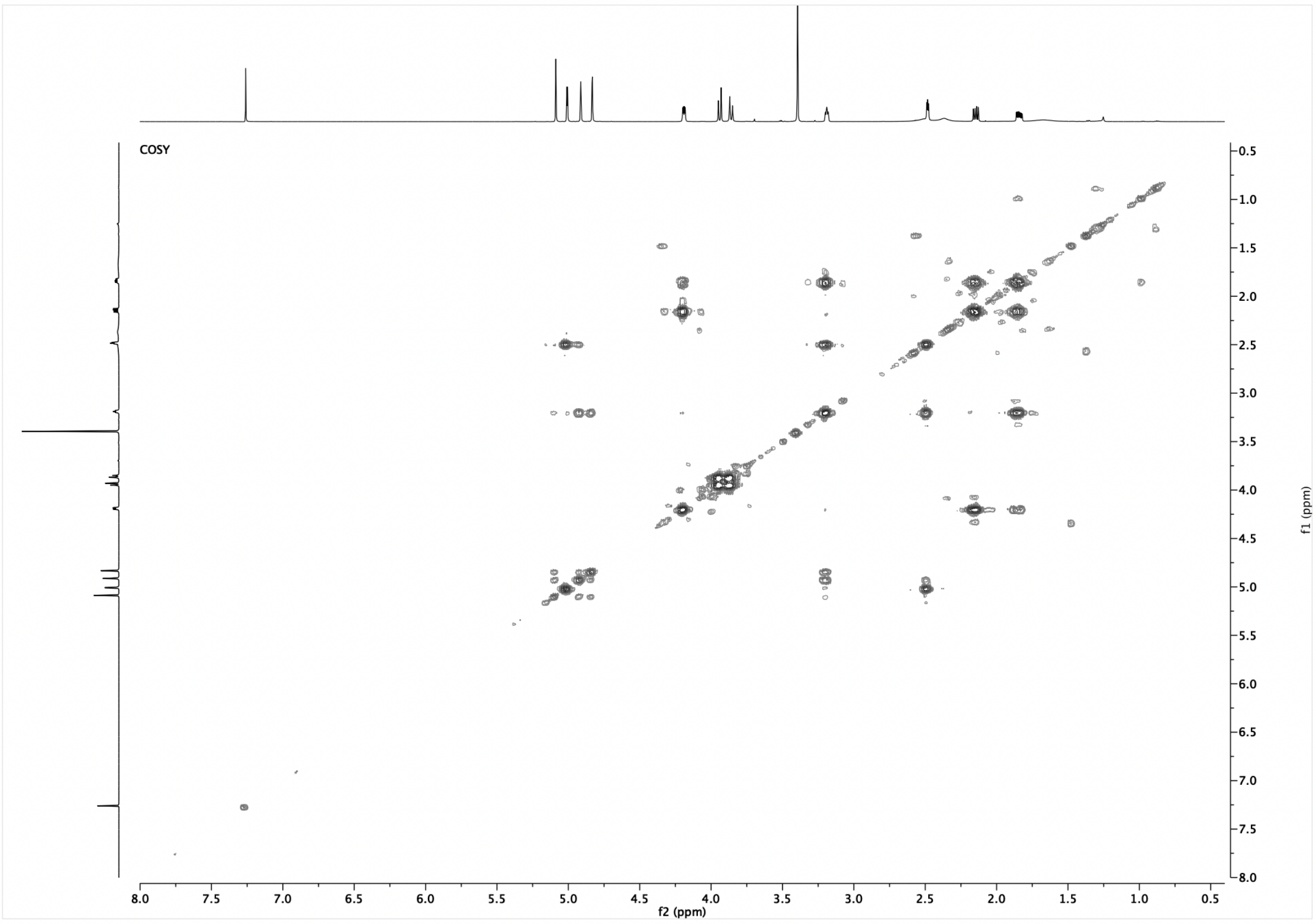

Figure S17. The ^{1}H-^{1}H COSY Spectrum of Compound **2** in $CDCl_3$

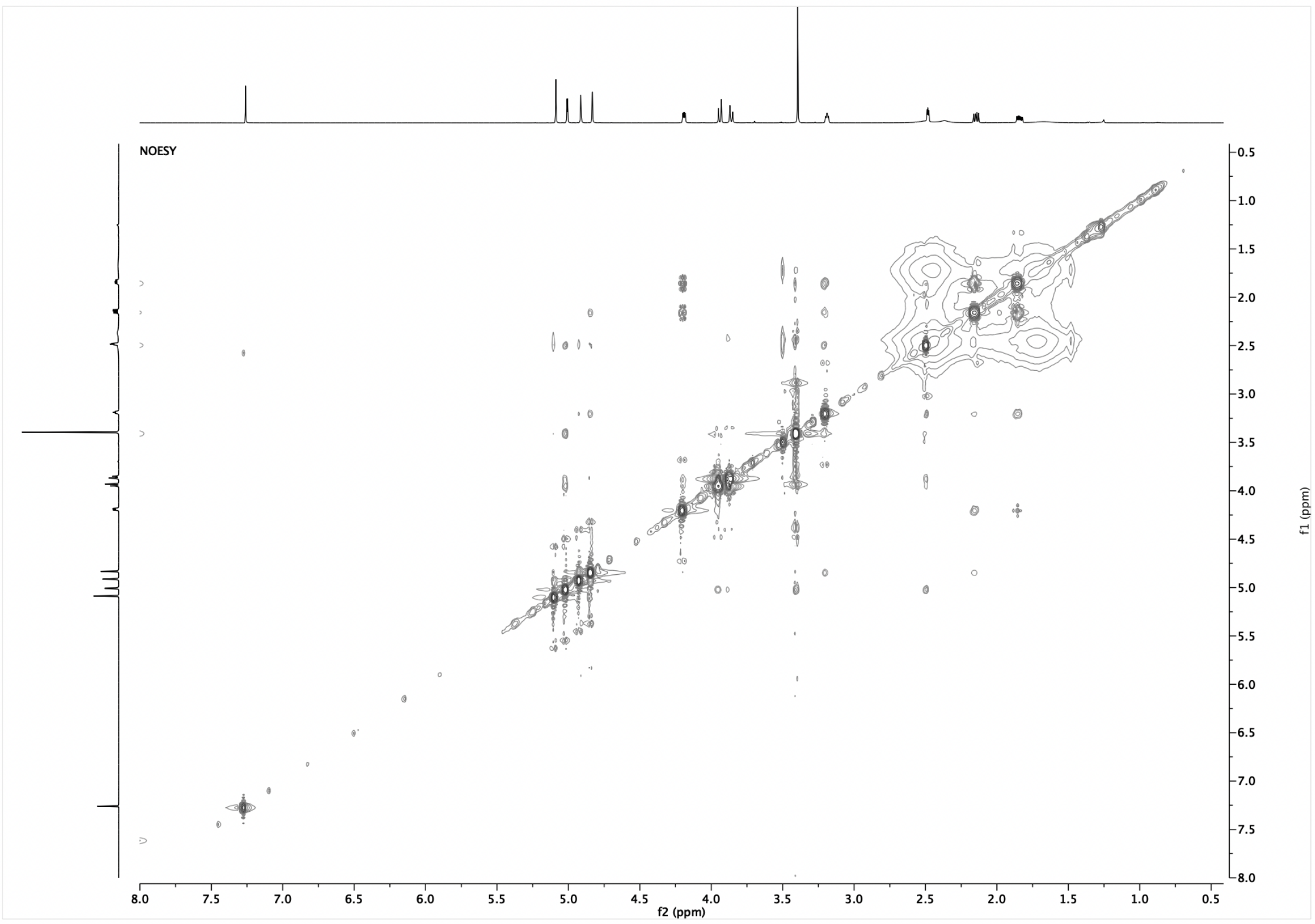

Figure S18. The NOESY Spectrum of Compound **2** in $CDCl_3$

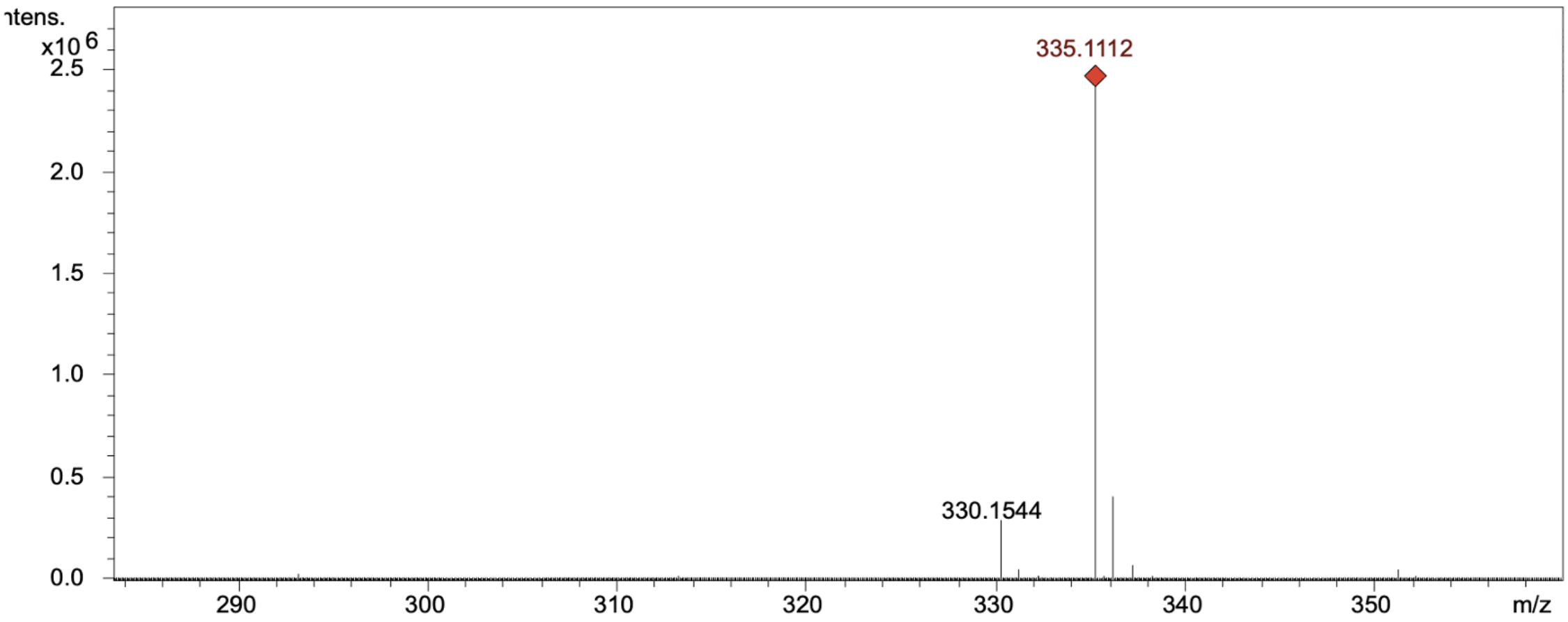

Meas. m/z	#	Formula	Score	m/z	err [mDa]	err [ppm]	mSigma	rdb	e⁻ Conf	N-Rule
335.1112	1	C 15 H 20 Na O 7	100	335.1101	-1.1	-3.2	1.3	5.5	even	ok

Figure S19. The (+)-HRESIMS Spectrum of Compound **3**

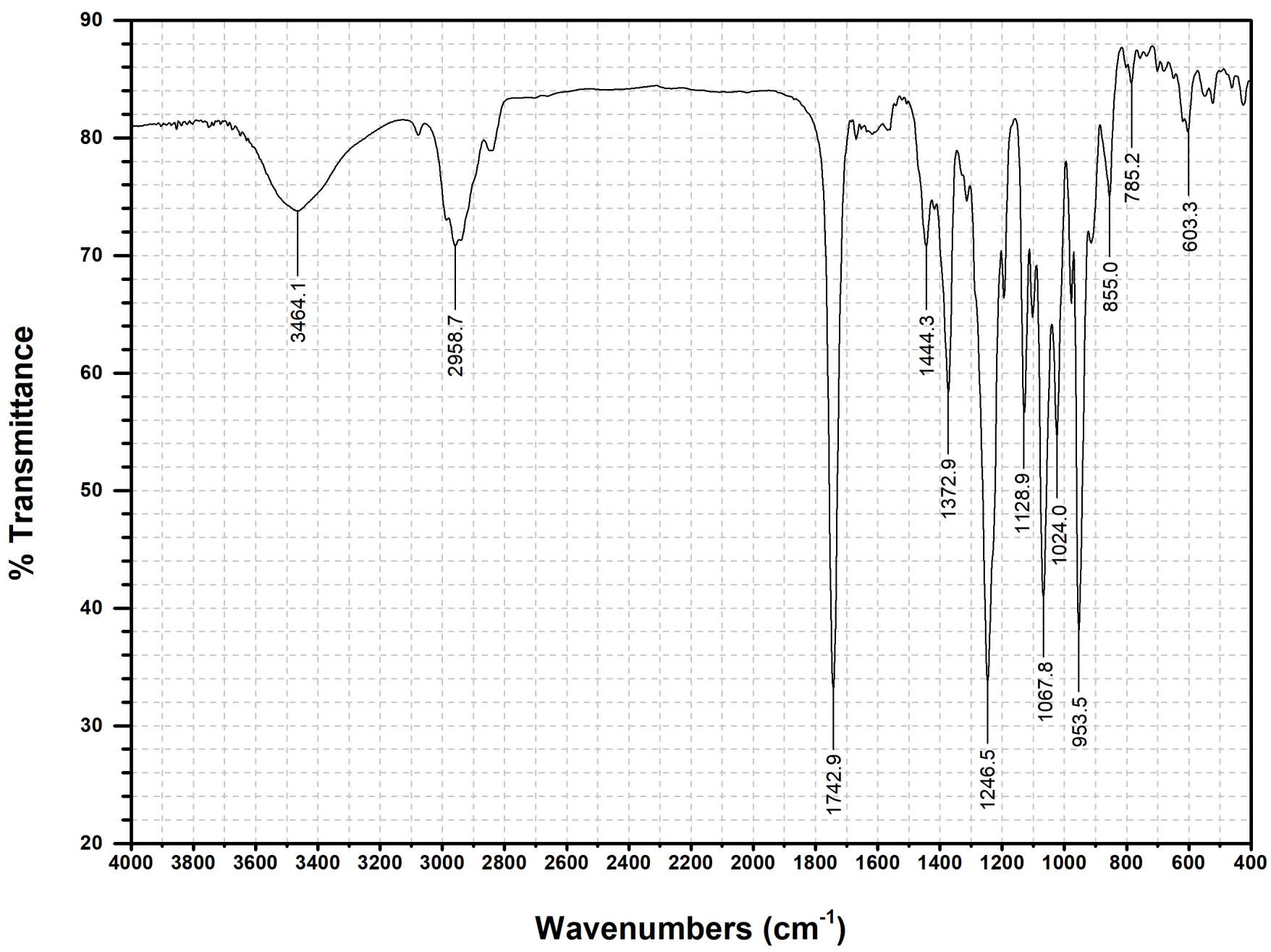

Figure S20. The IR Spectrum of Compound **3**

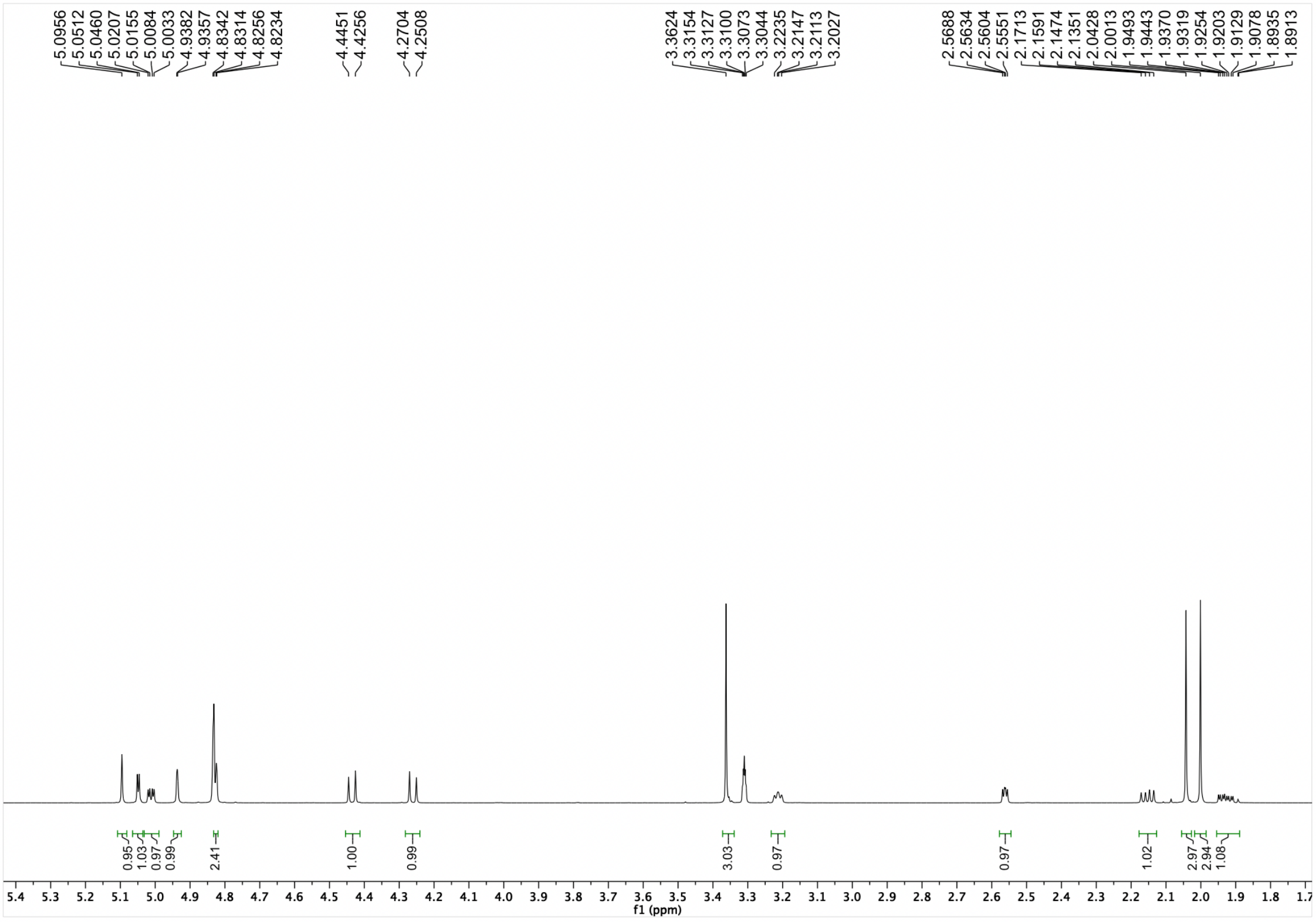

Figure S21. The ^{1}H NMR Spectrum of Compound **3** in CD_3OD

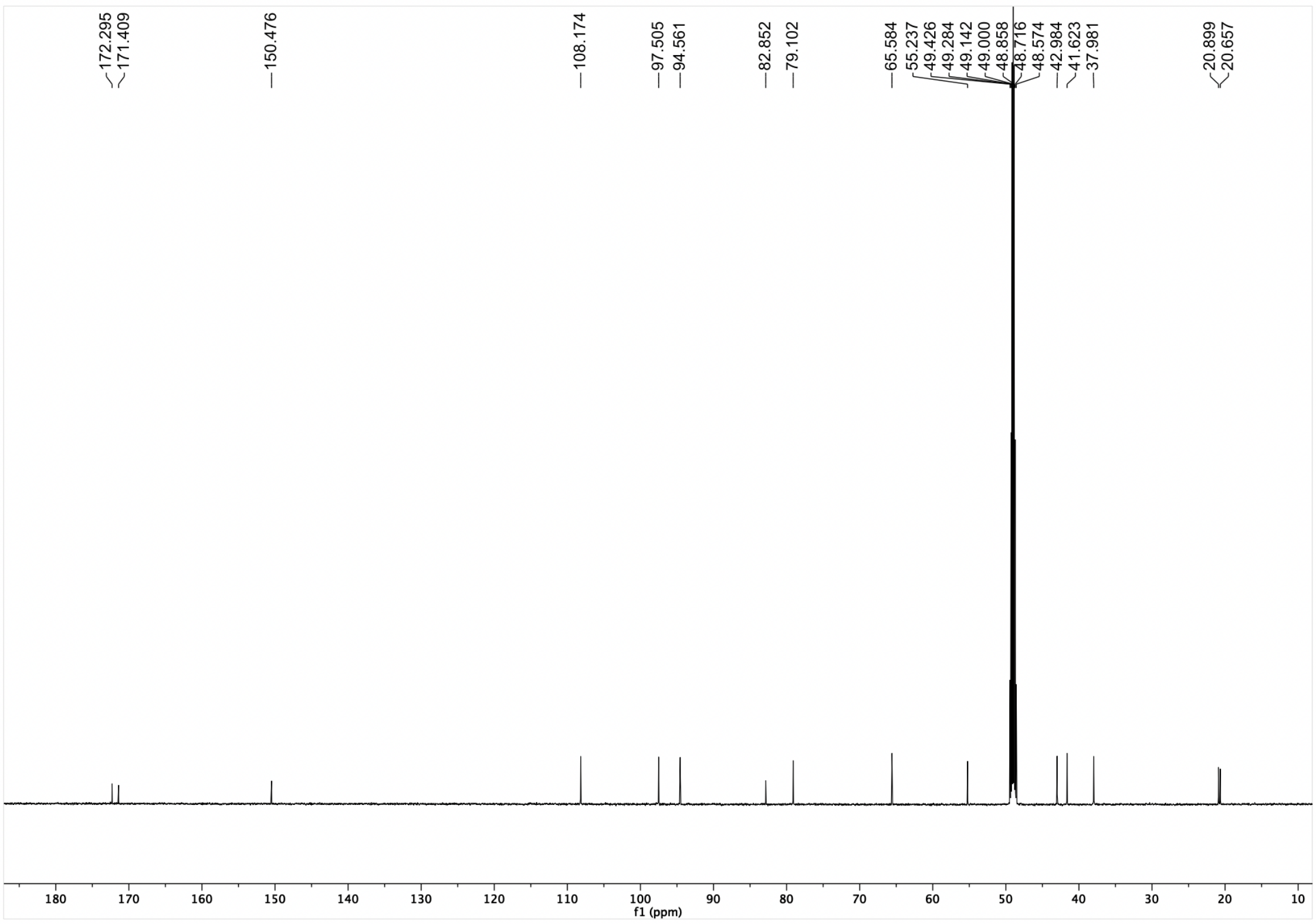

Figure S22. The ^{13}C NMR Spectrum of Compound **3** in CD_3OD

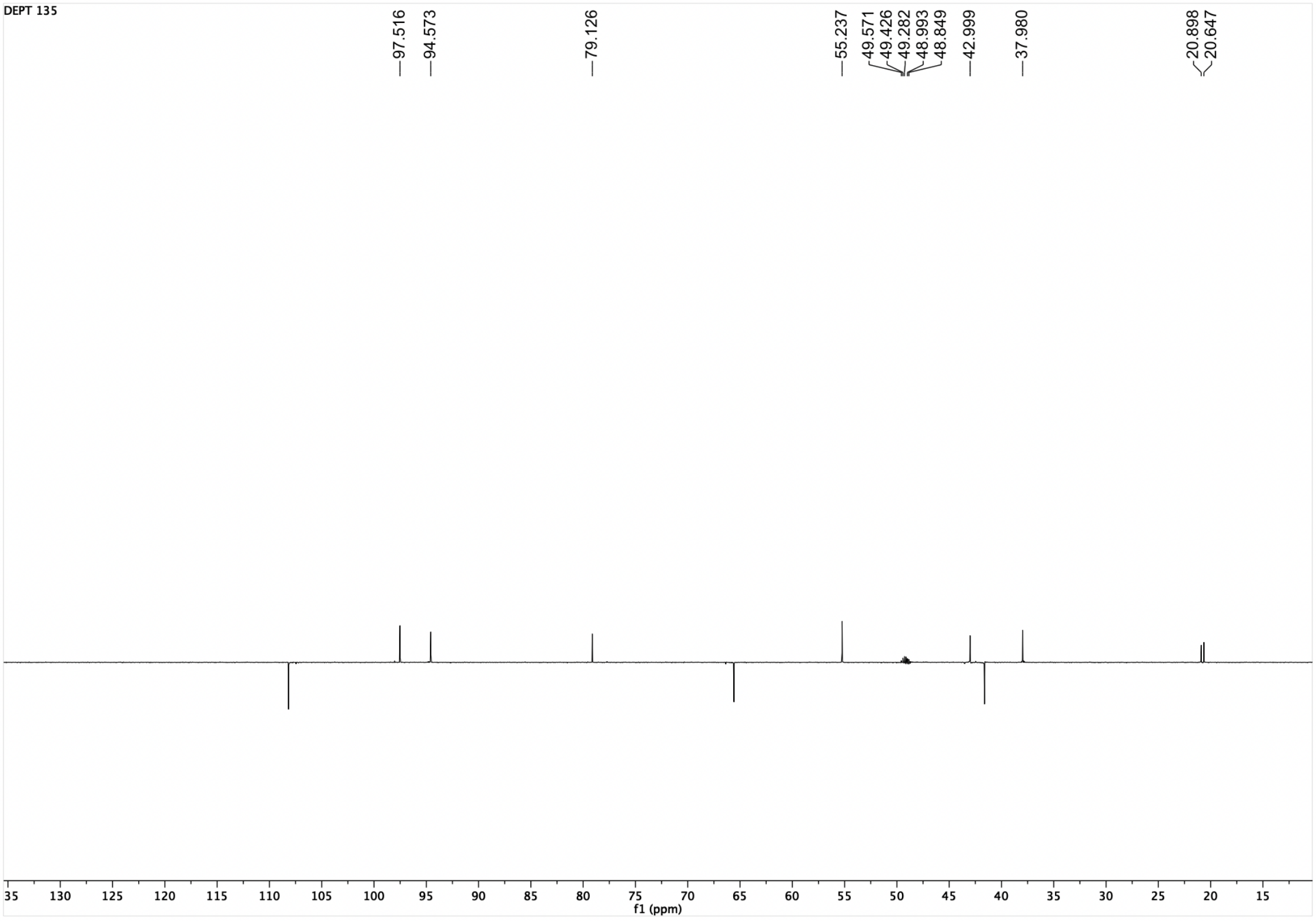

Figure S23. The DEPT 135 Spectrum of Compound **3** in CD_3OD

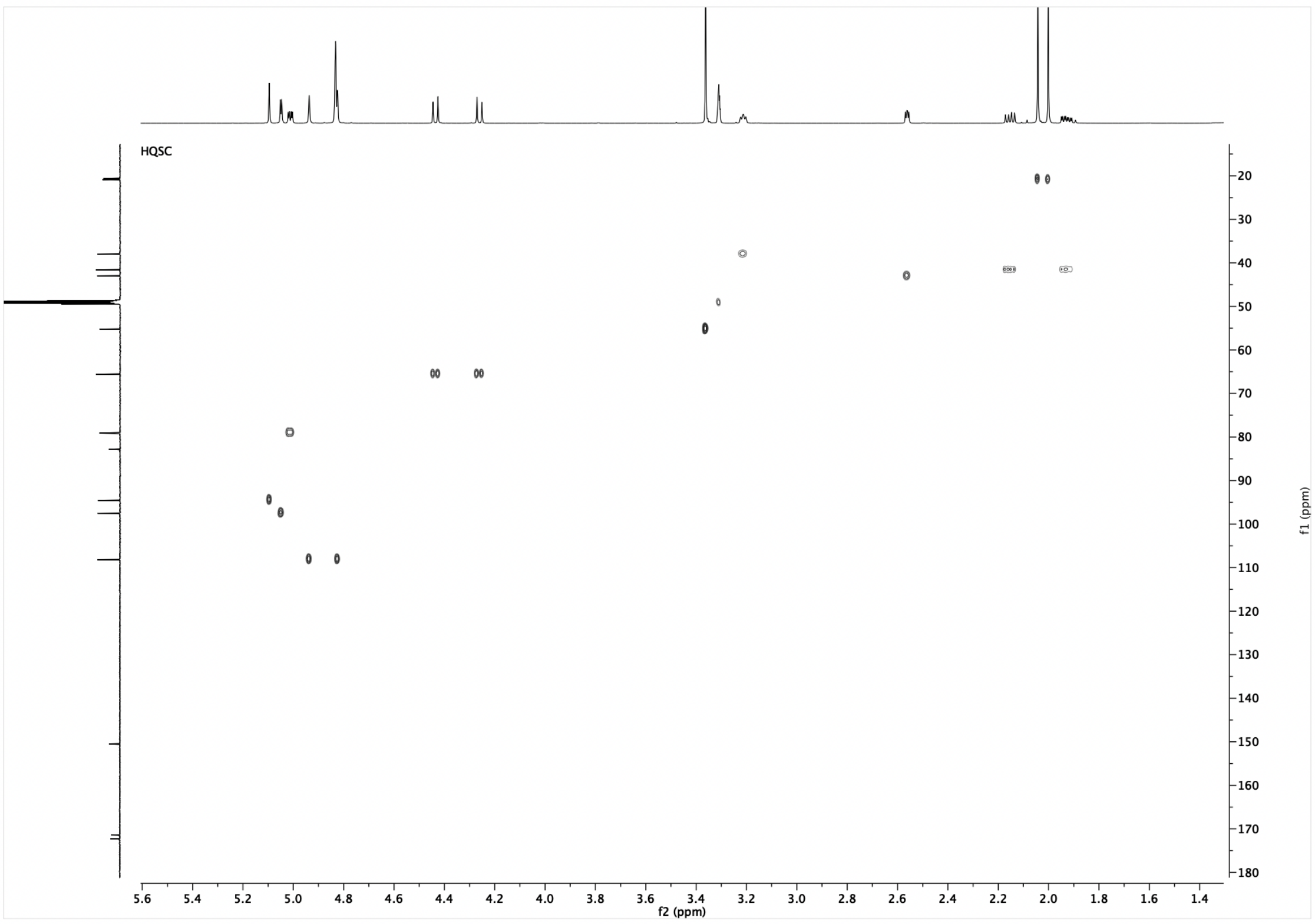

Figure S24. The HSQC Spectrum of Compound **3** in CD_3OD

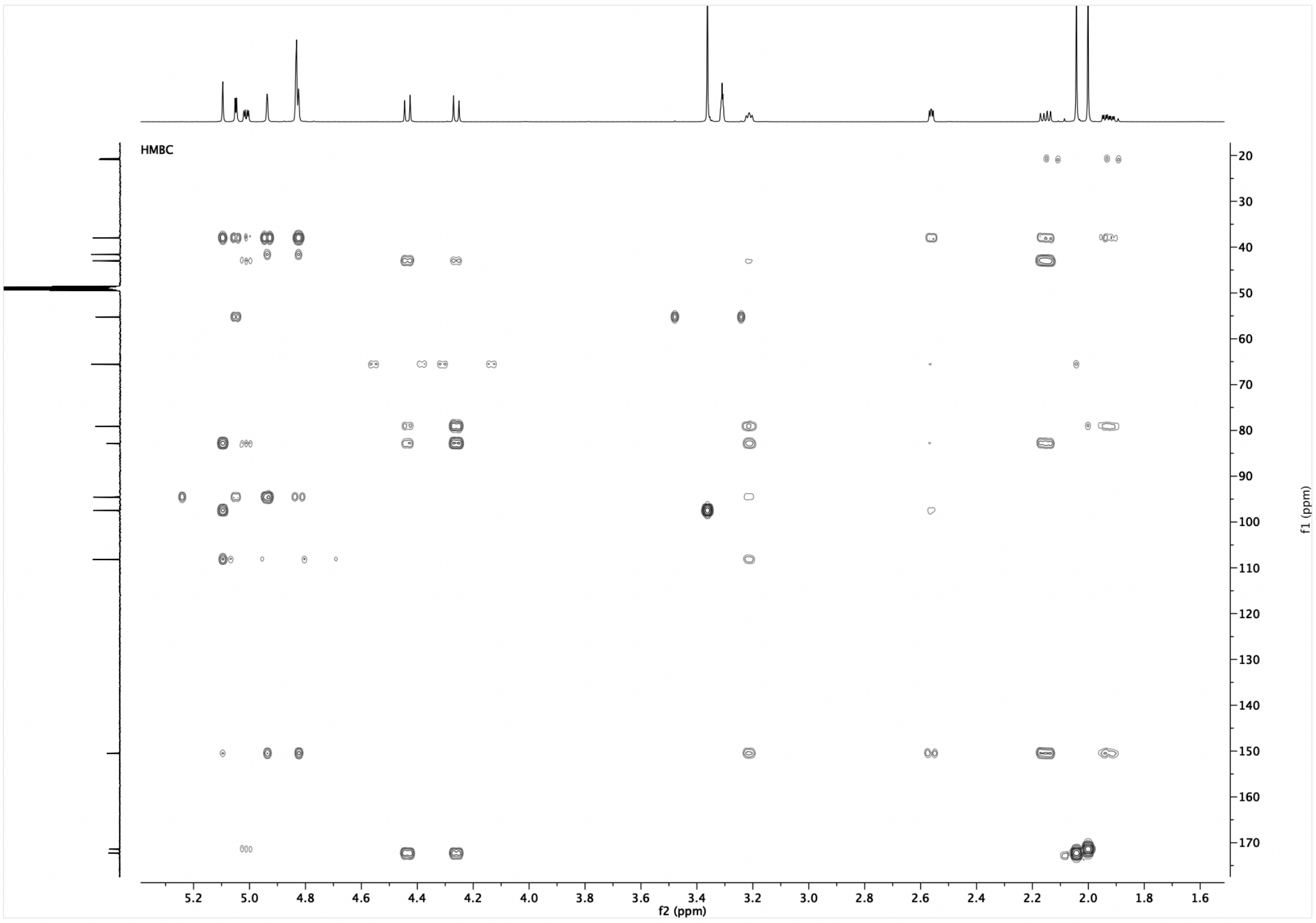

Figure S25. The HMBC Spectrum of Compound **3** in CD_3OD

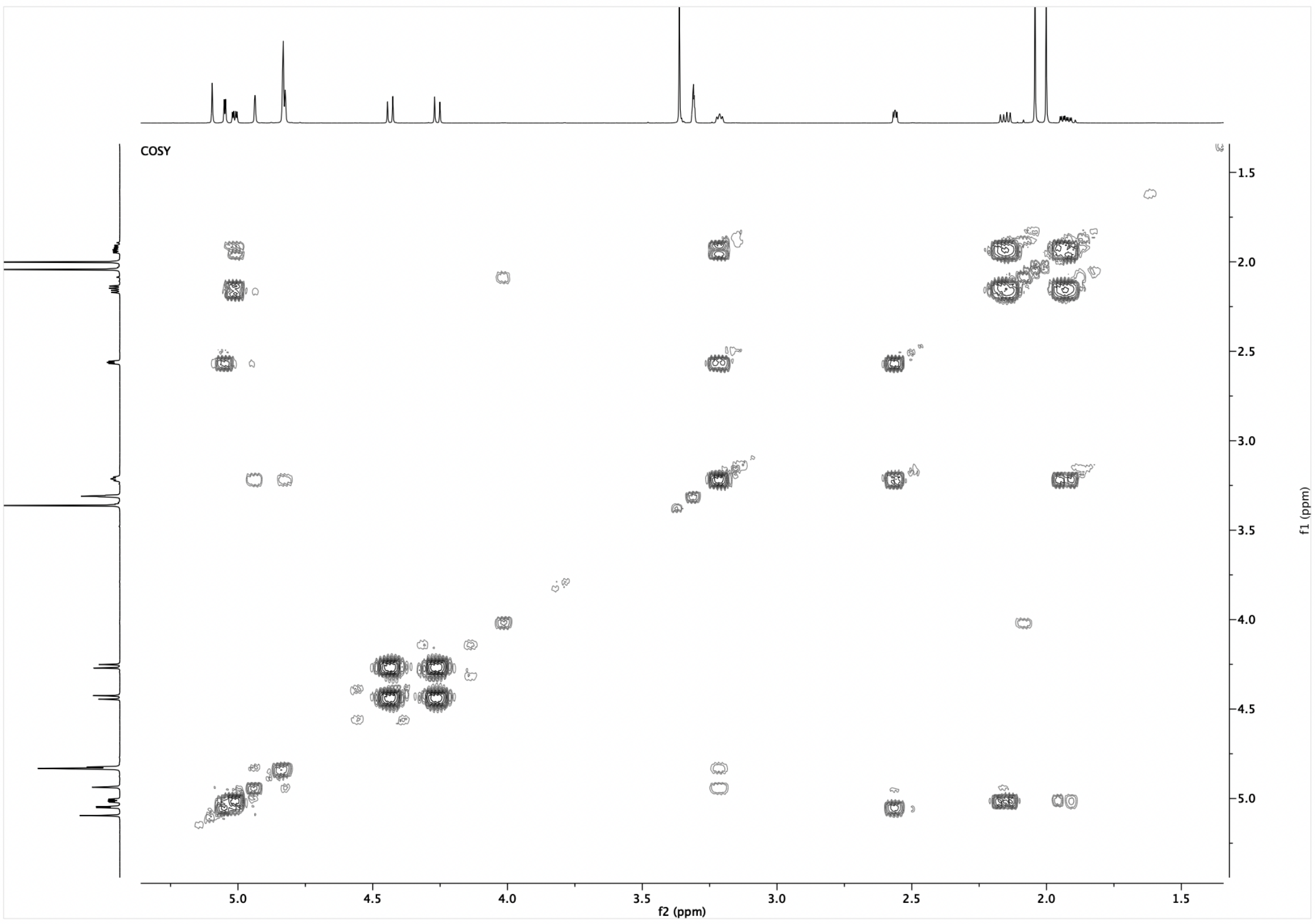

Figure S26. The ^{1}H-^{1}H COSY Spectrum of Compound **3** in CD_3OD

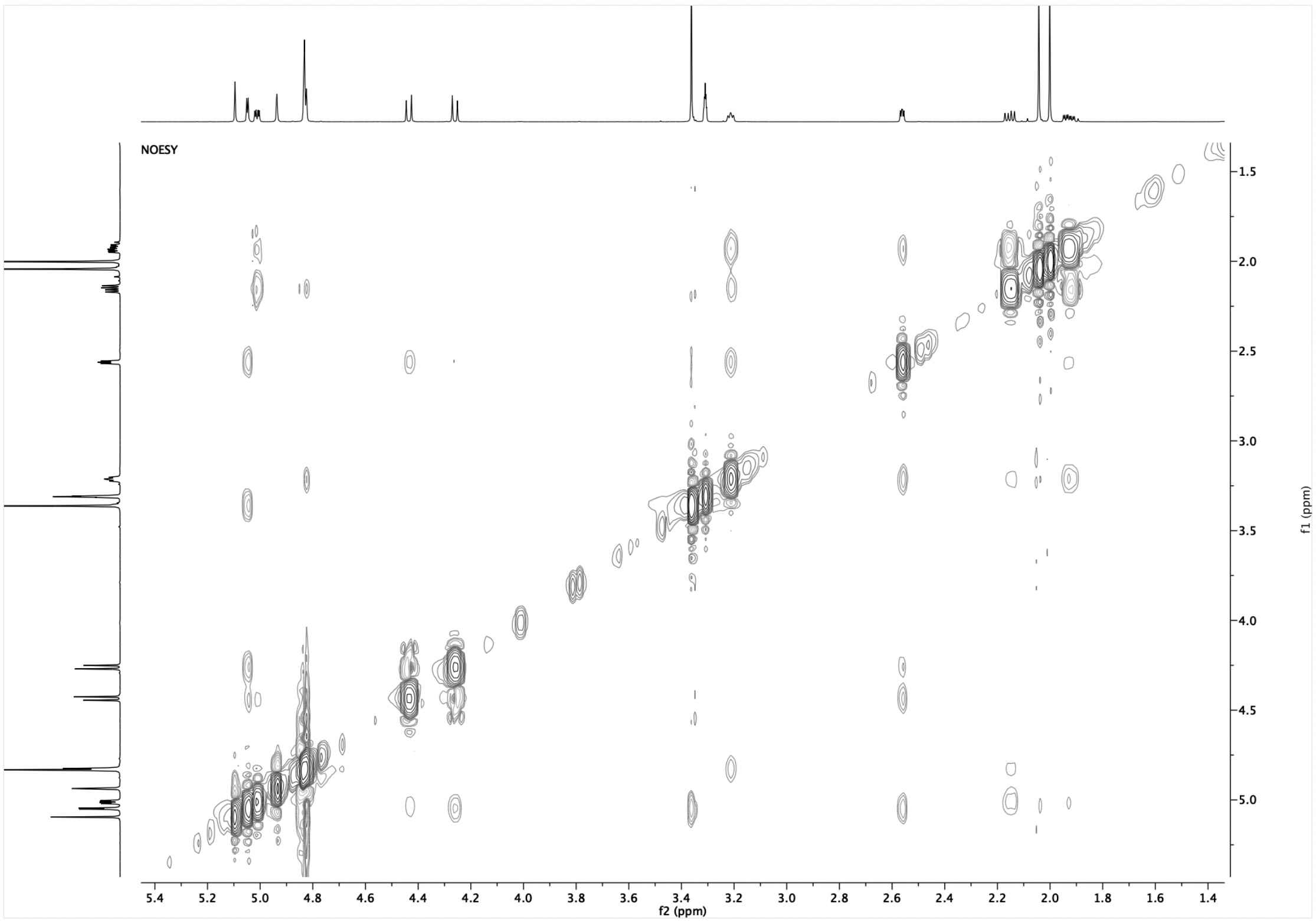

Figure S27. The NOESY Spectrum of Compound **3** in CD_3OD

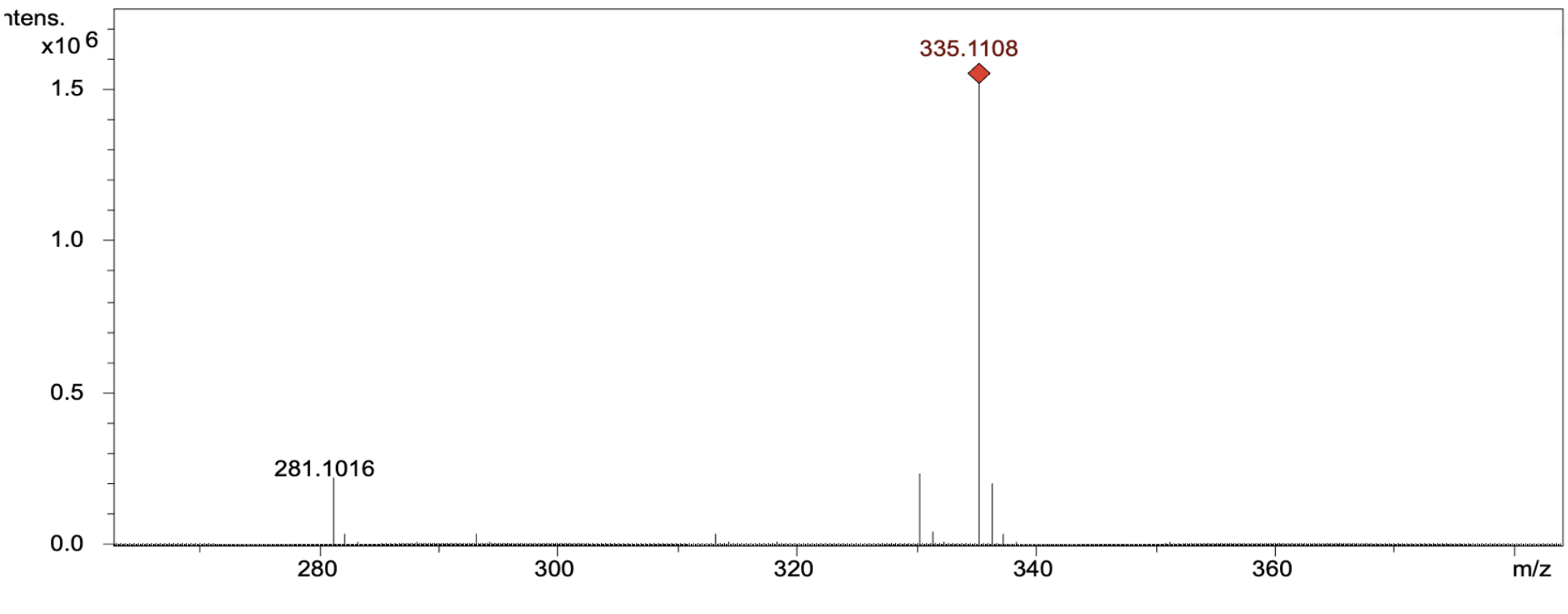

Meas. m/z	#	Formula	Score	m/z	err [mDa]	err [ppm]	mSigma	rdb	e⁻ Conf	N-Rule
335.1108	1	C 15 H 20 Na O 7	100	335.1101	-0.7	-2.1	17.2	5.5	even	ok

Figure S28. The (+)-HRESIMS Spectrum of Compound **4**

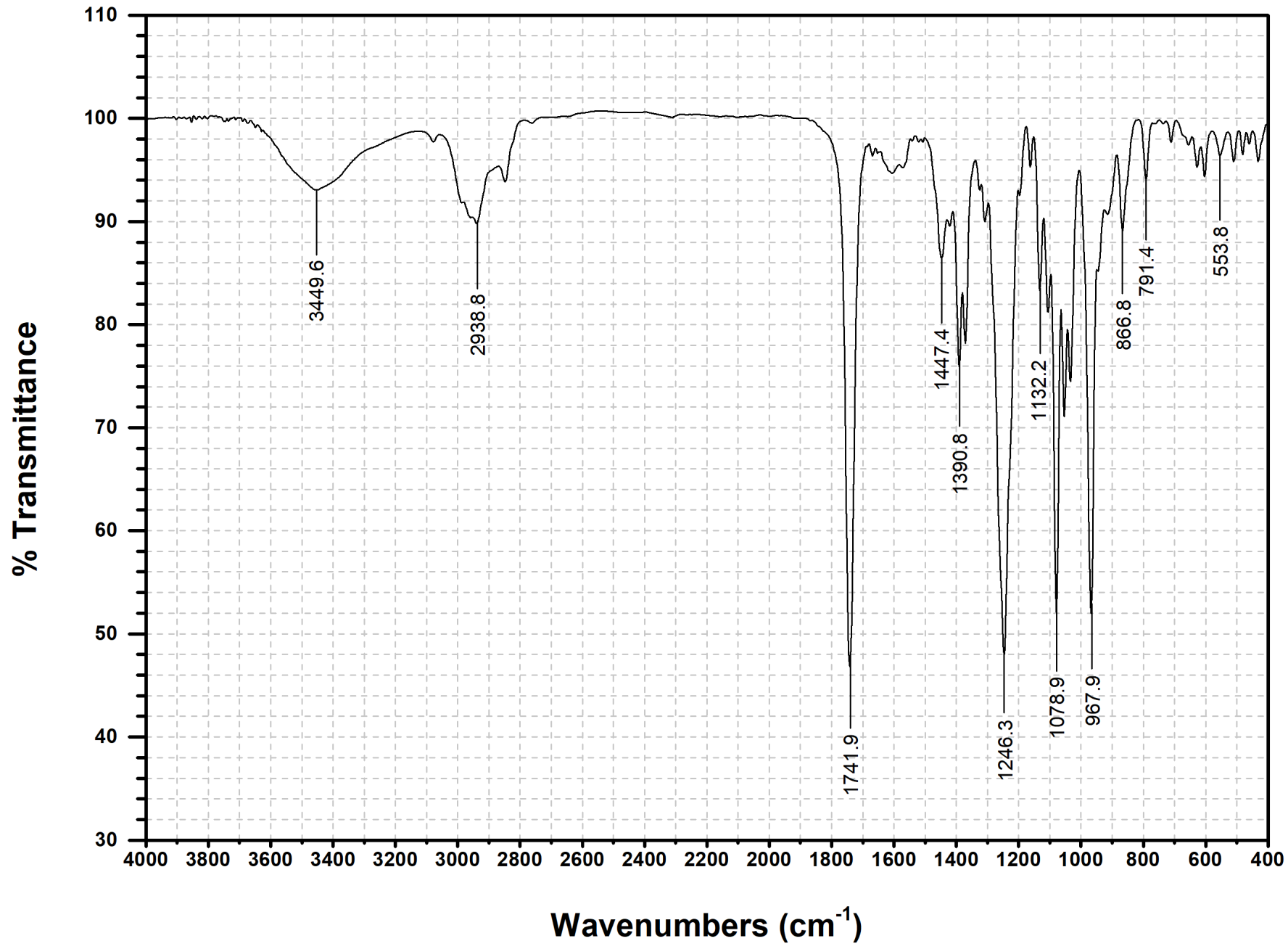

Figure S29. The IR Spectrum of Compound **4**

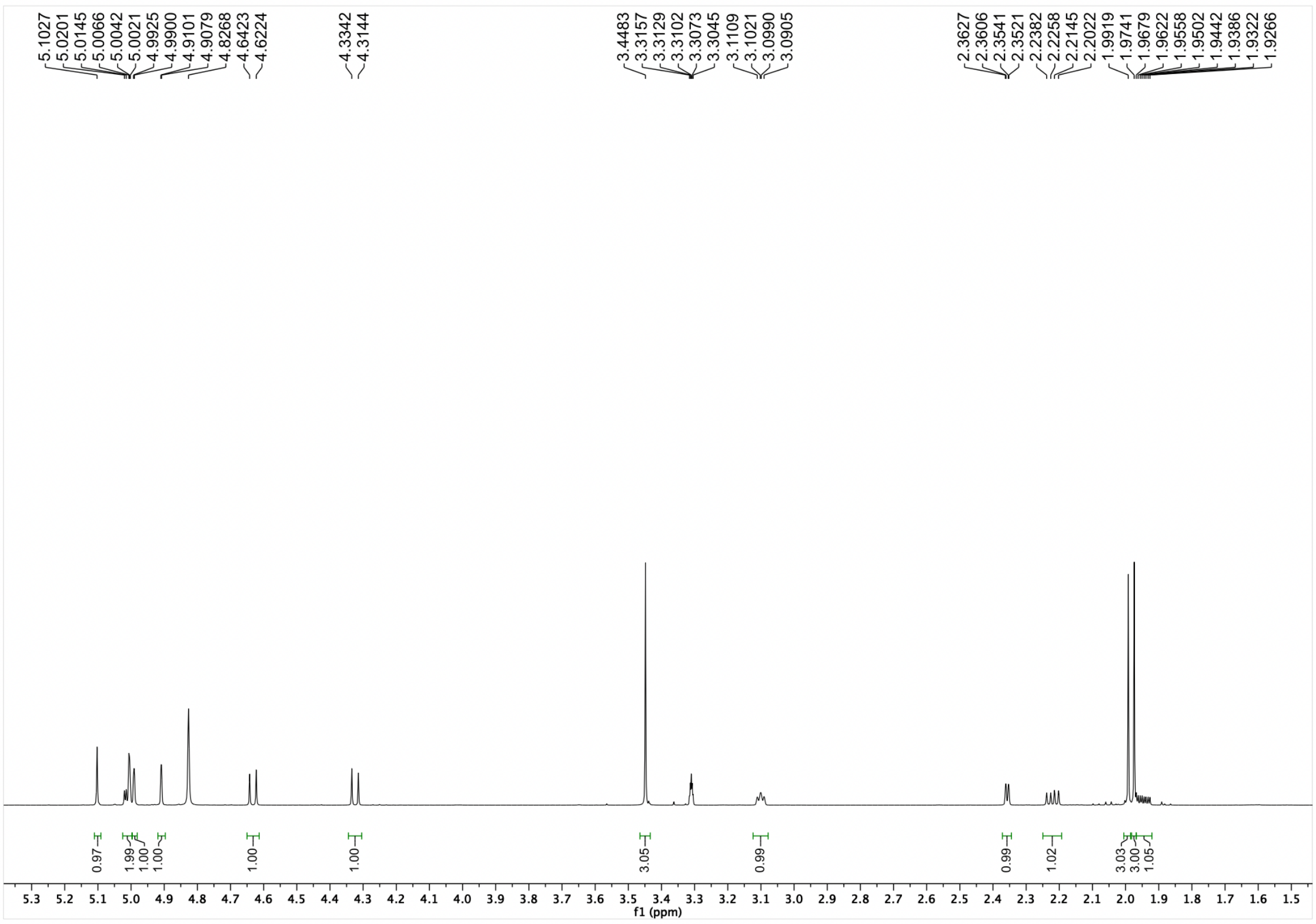

Figure S30. The ^{1}H NMR Spectrum of Compound **4** in CD_3OD

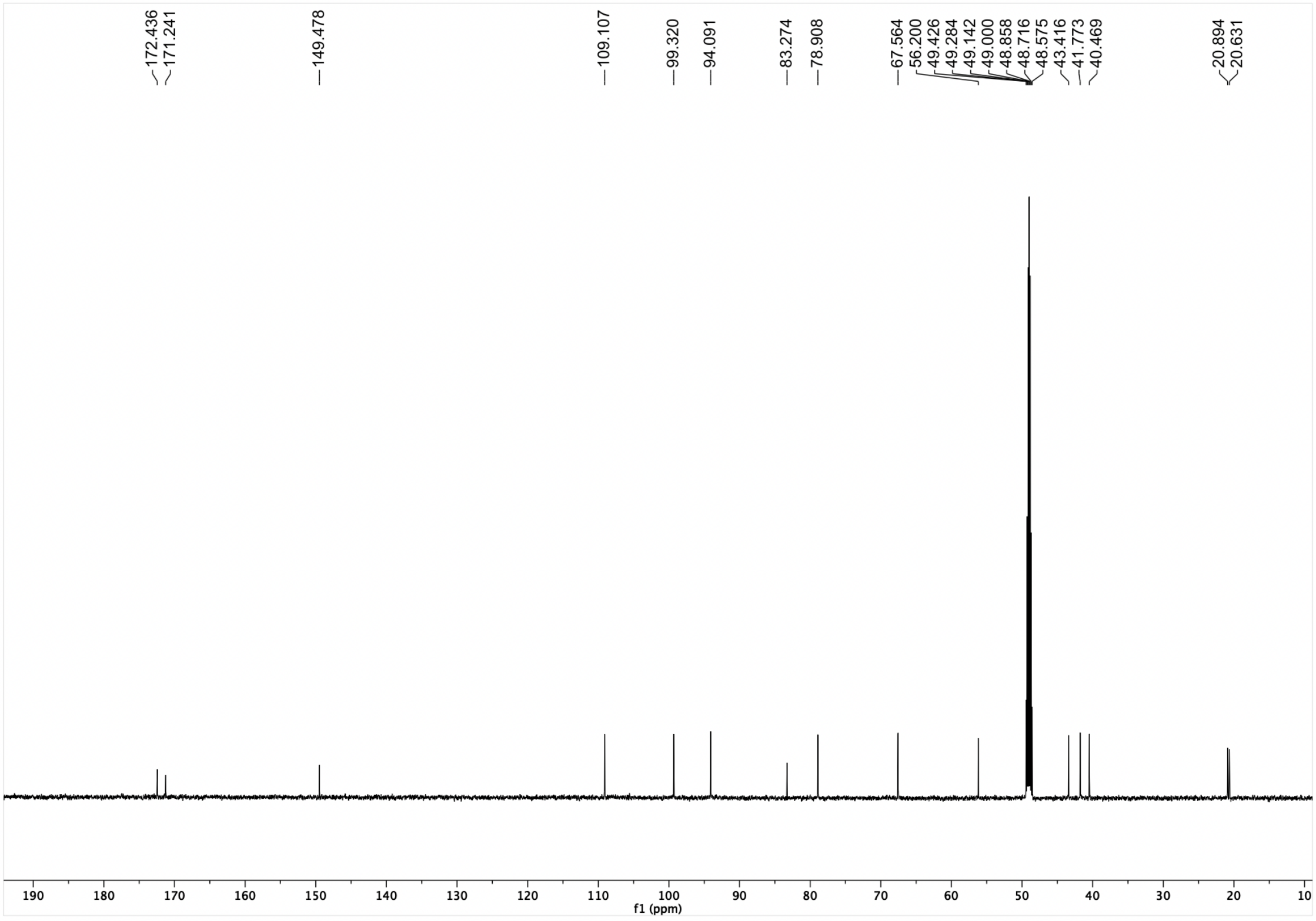

Figure S31. The ^{13}C NMR Spectrum of Compound **4** in CD_3OD

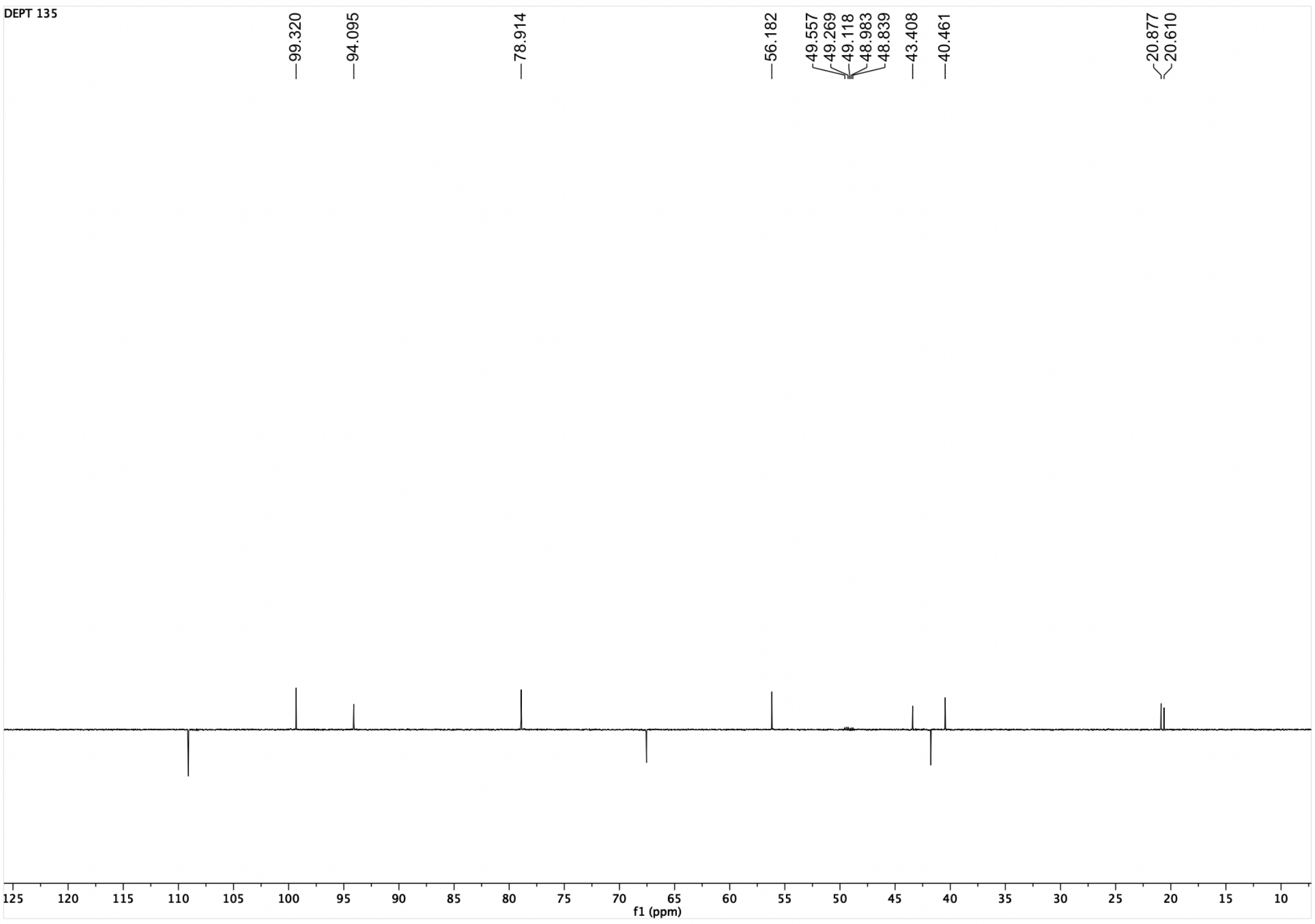

Figure S32. The DEPT 135 Spectrum of Compound **4** in CD_3OD

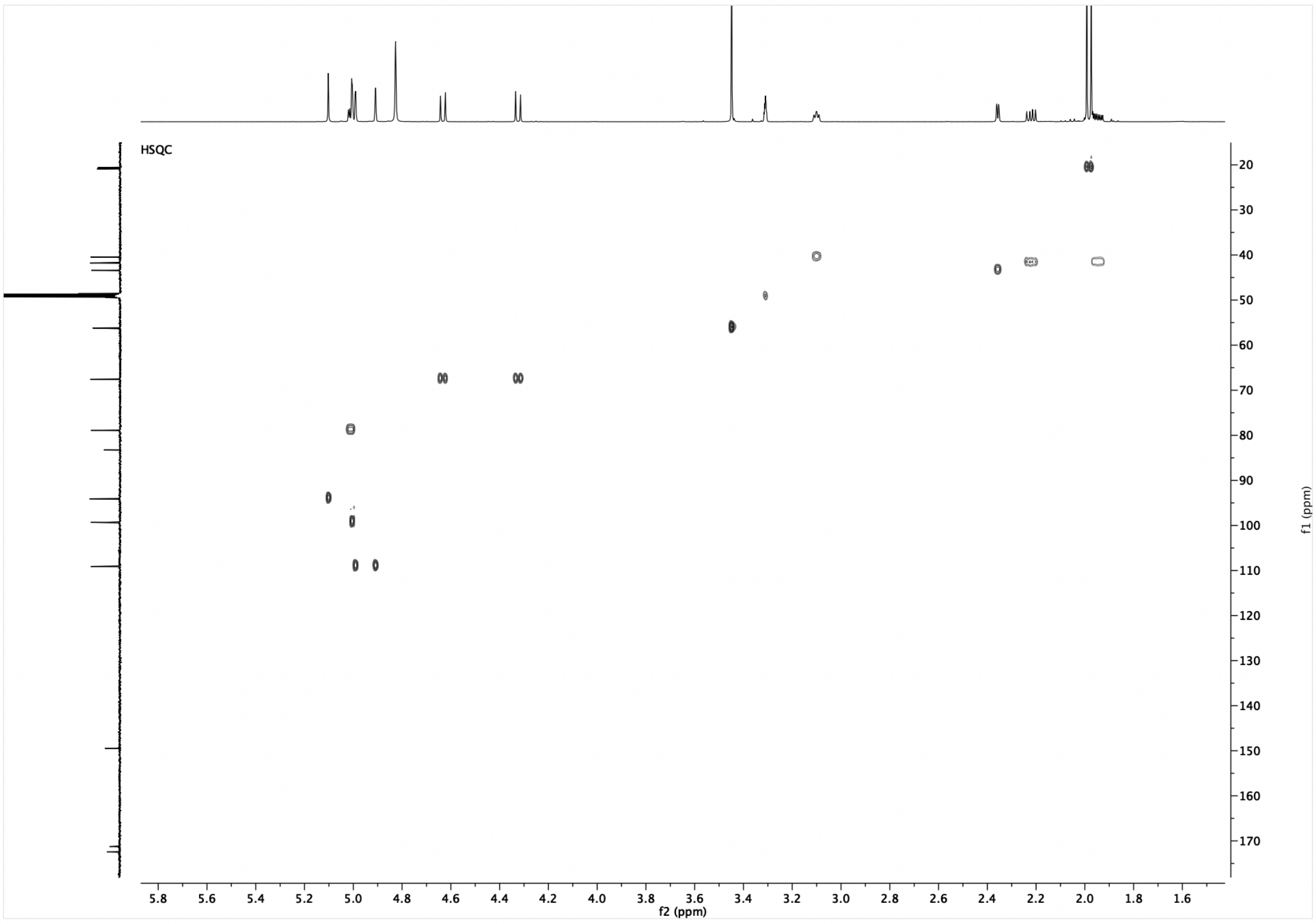

Figure S33. The HSQC Spectrum of Compound **4** in CD_3OD

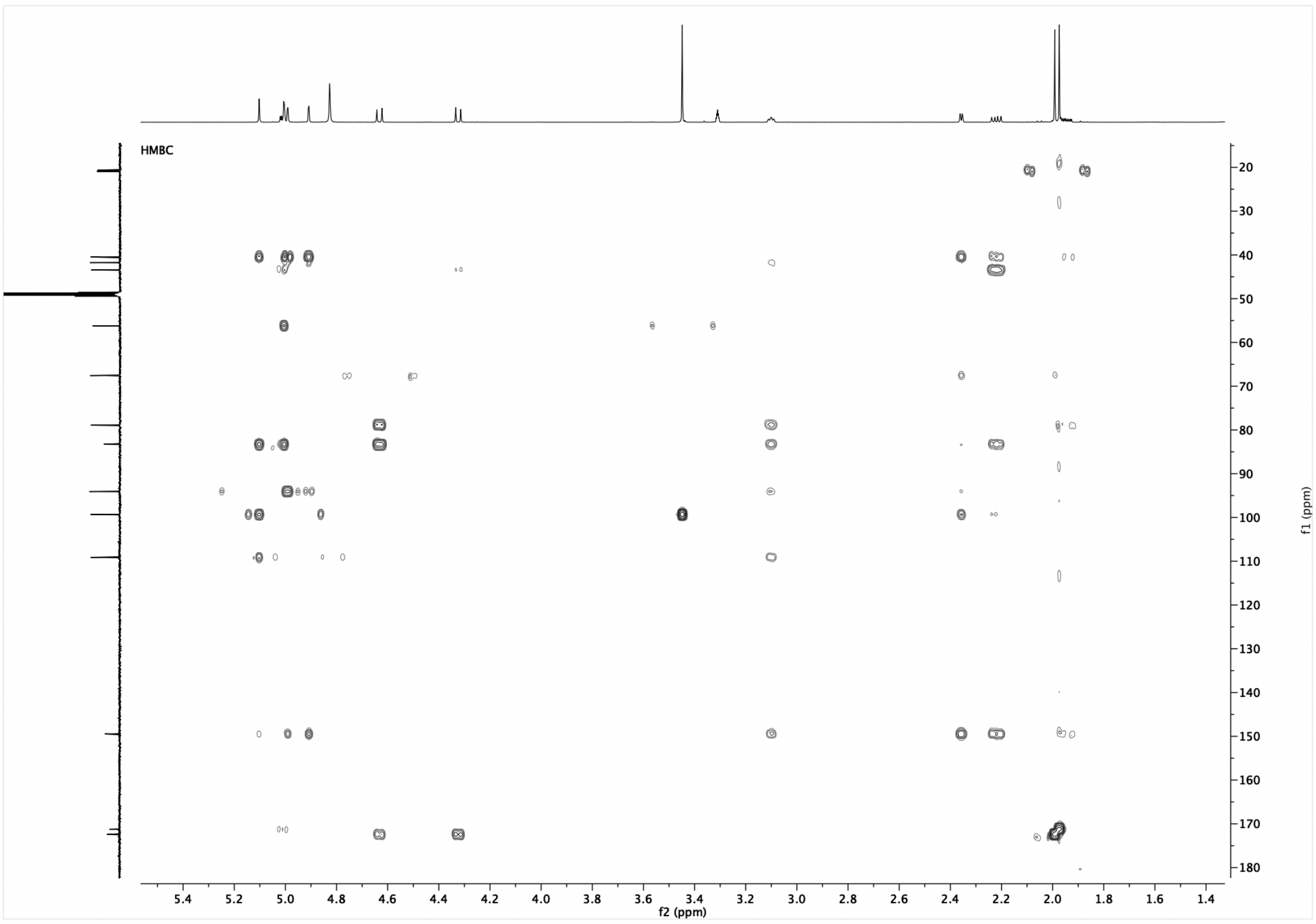

Figure S34. The HMBC Spectrum of Compound **4** in CD_3OD

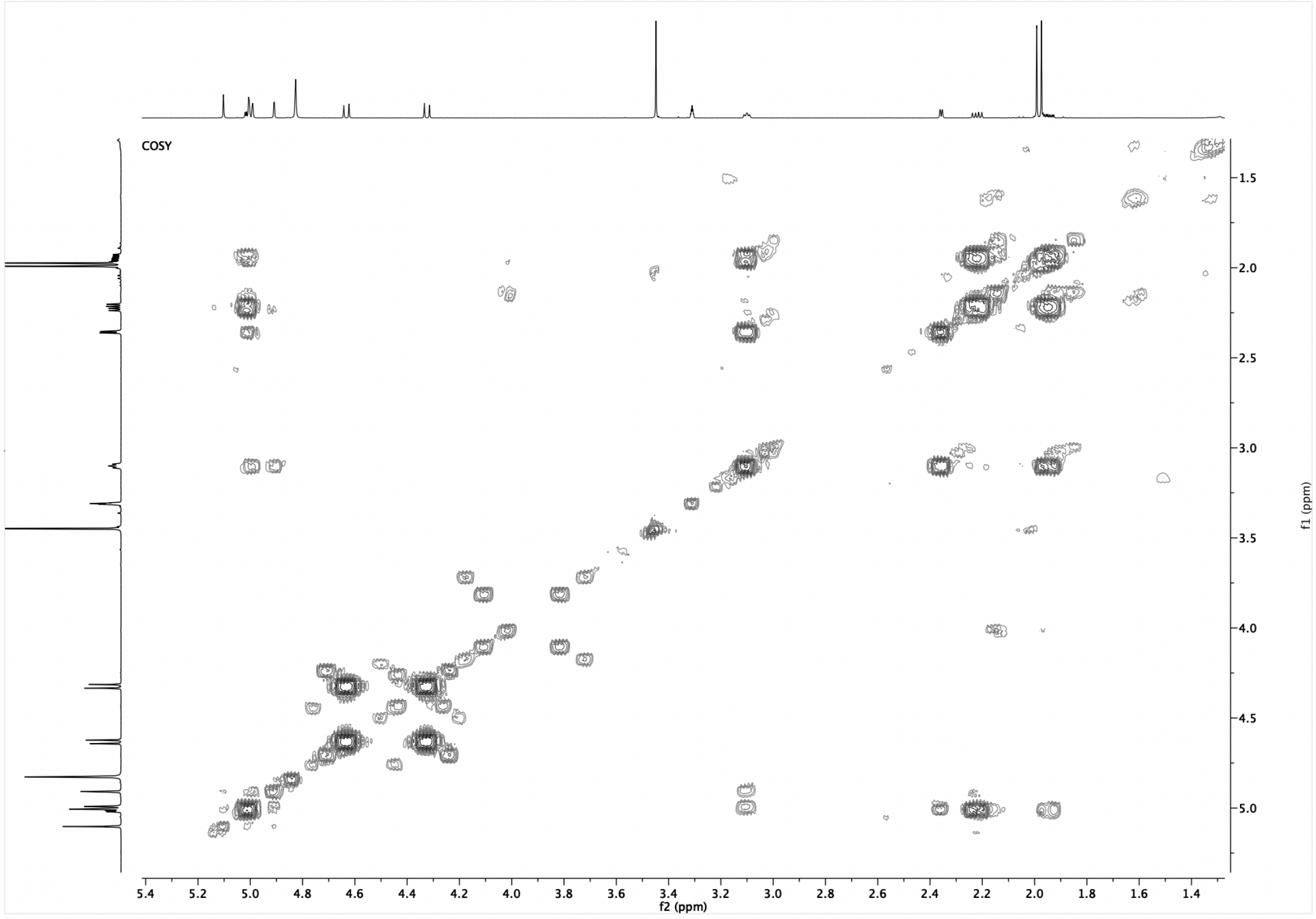

Figure S35. The ^{1}H-^{1}H COSY Spectrum of Compound **4** in CD_3OD

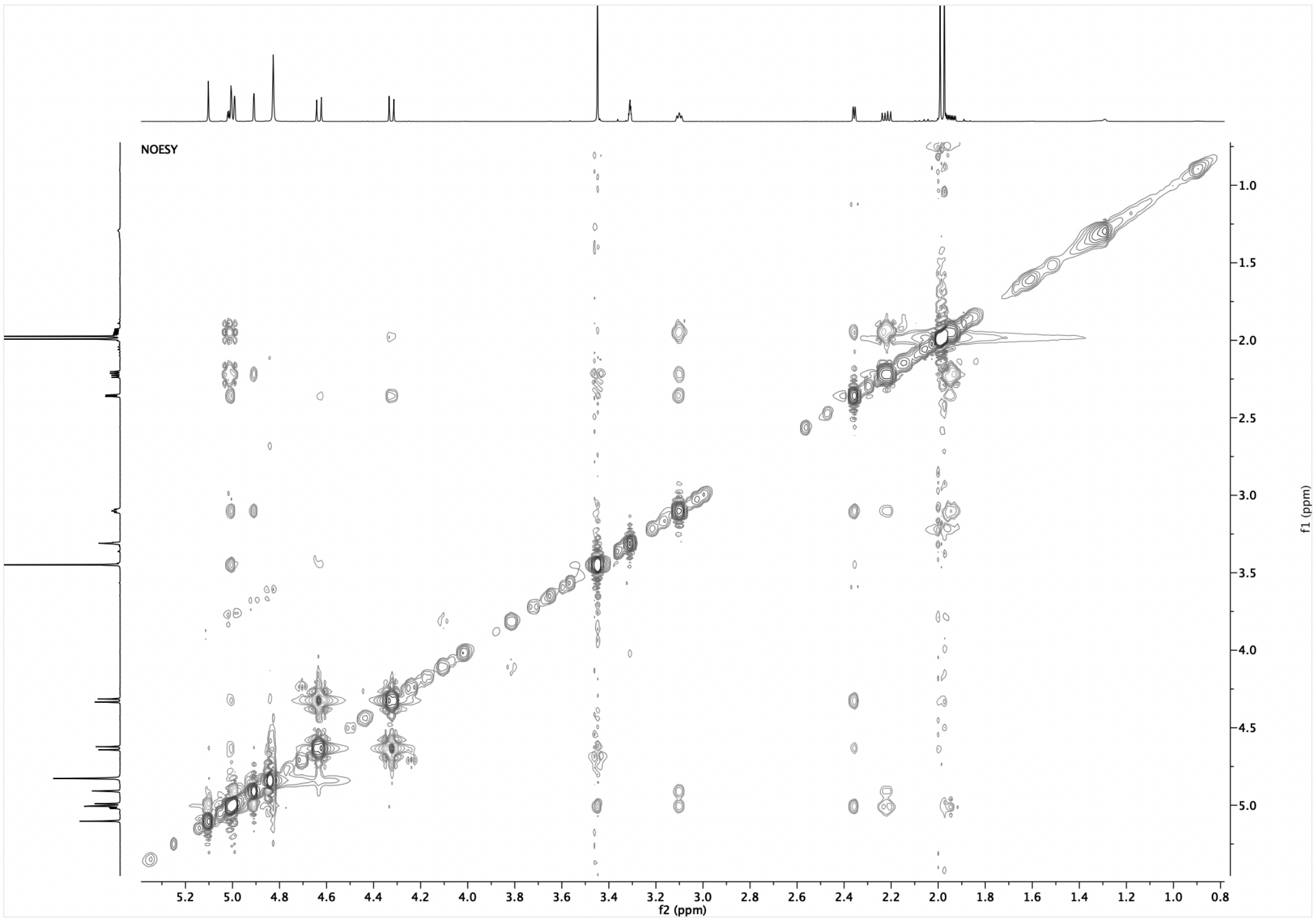

Figure S36. The NOESY Spectrum of Compound **4** in CD_3OD

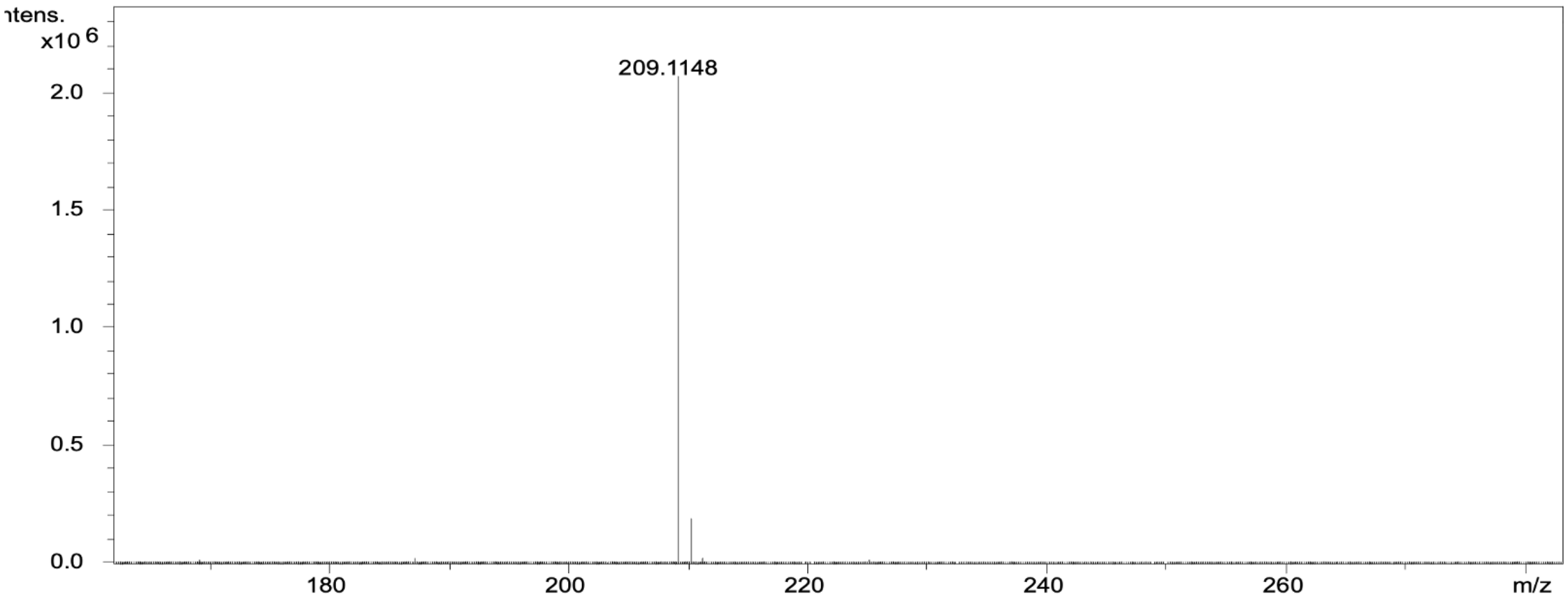

Meas. m/z	#	Formula	Score	m/z	err [mDa]	err [ppm]	mSigma	rdb	e⁻ Conf	N-Rule
209.1148	1	C 10 H 18 Na O 3	100	209.1148	0	-0.1	11.1	1.5	even	ok

Figure S37. The (+)-HRESIMS Spectrum of Compound **5**

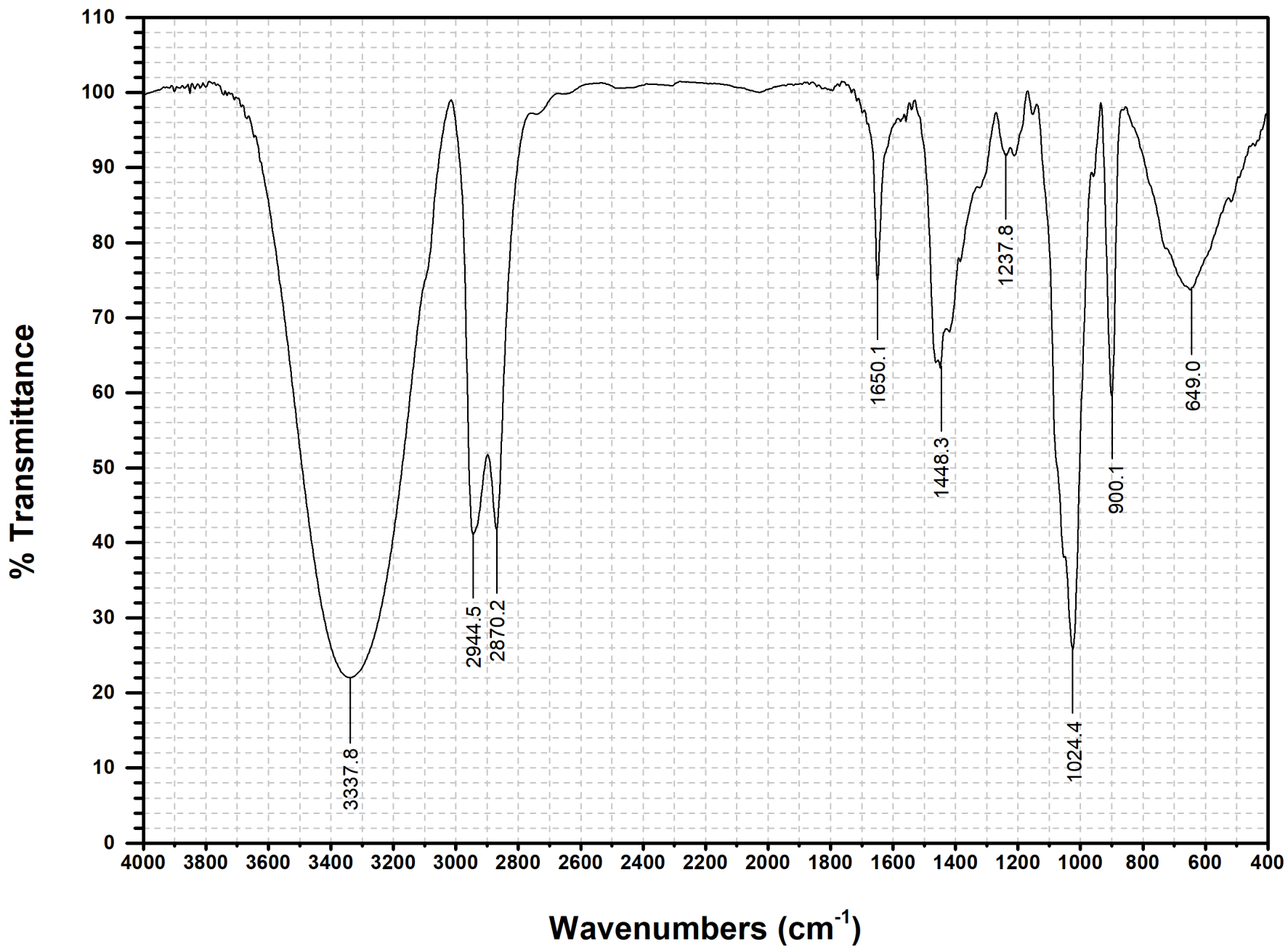

Figure S38. The IR Spectrum of Compound **5**

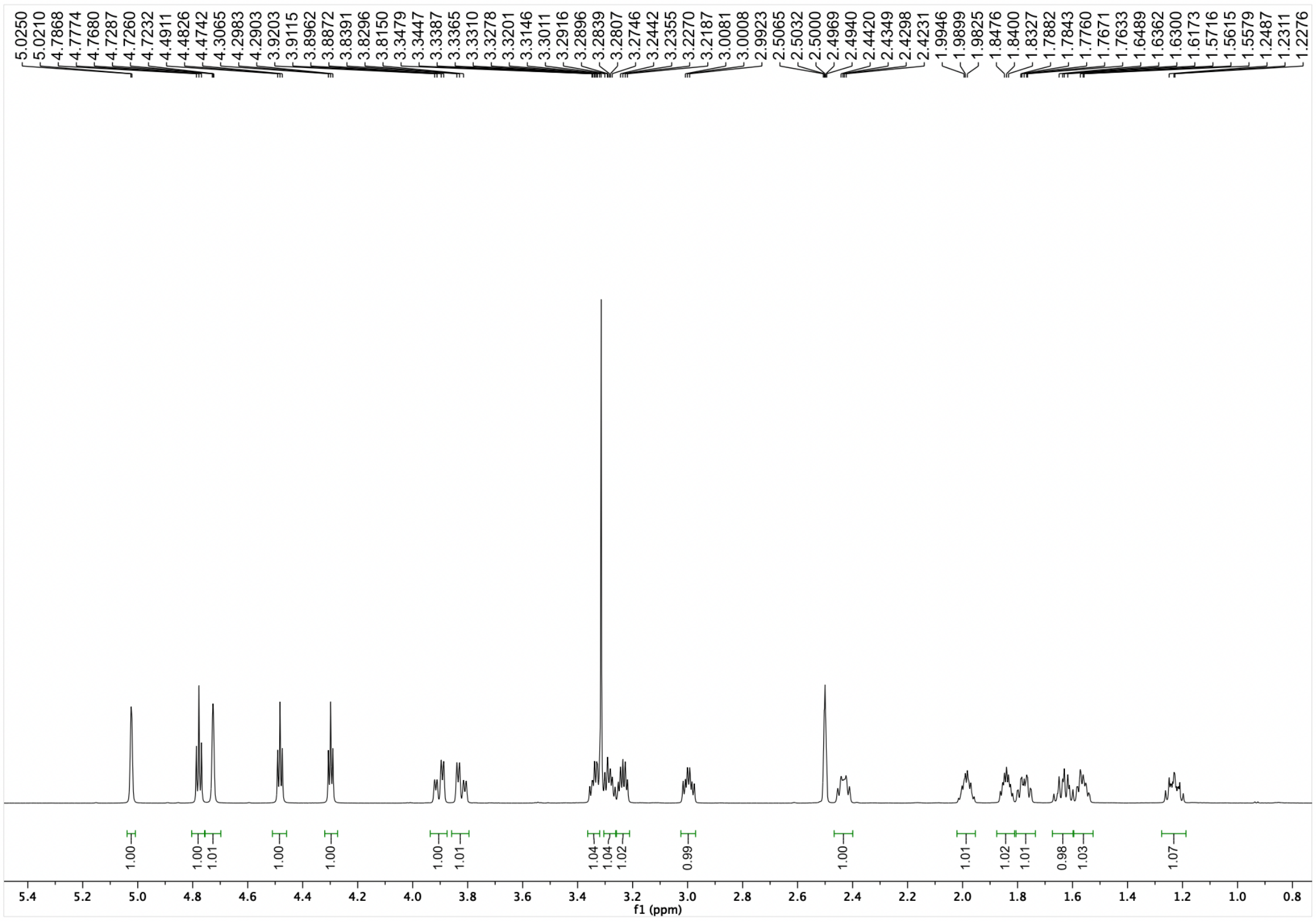

Figure S39. The ^{1}H NMR Spectrum of Compound **5** in DMSO-d_6

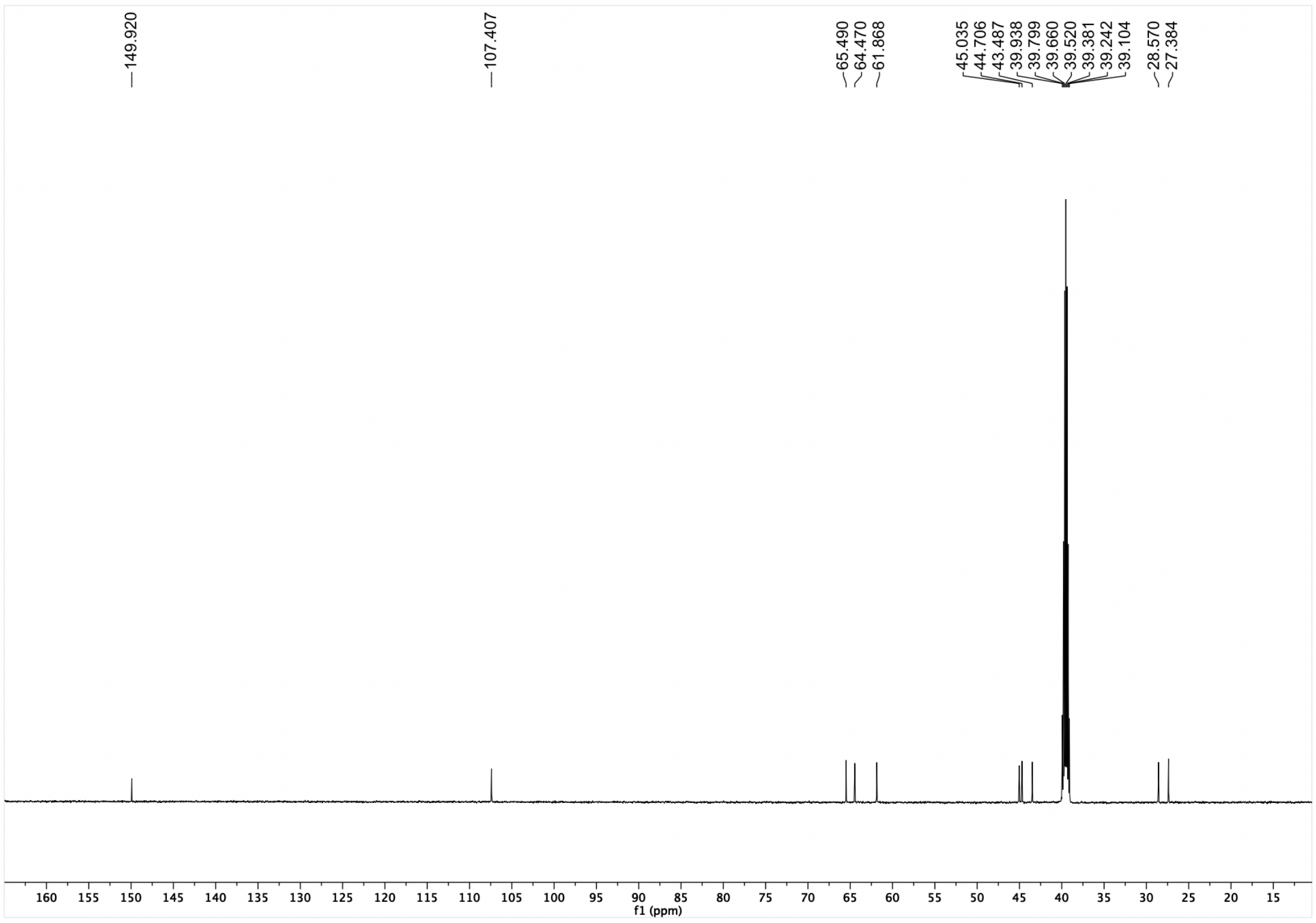

Figure S40. The ^{13}C NMR Spectrum of Compound **5** in DMSO-d_6

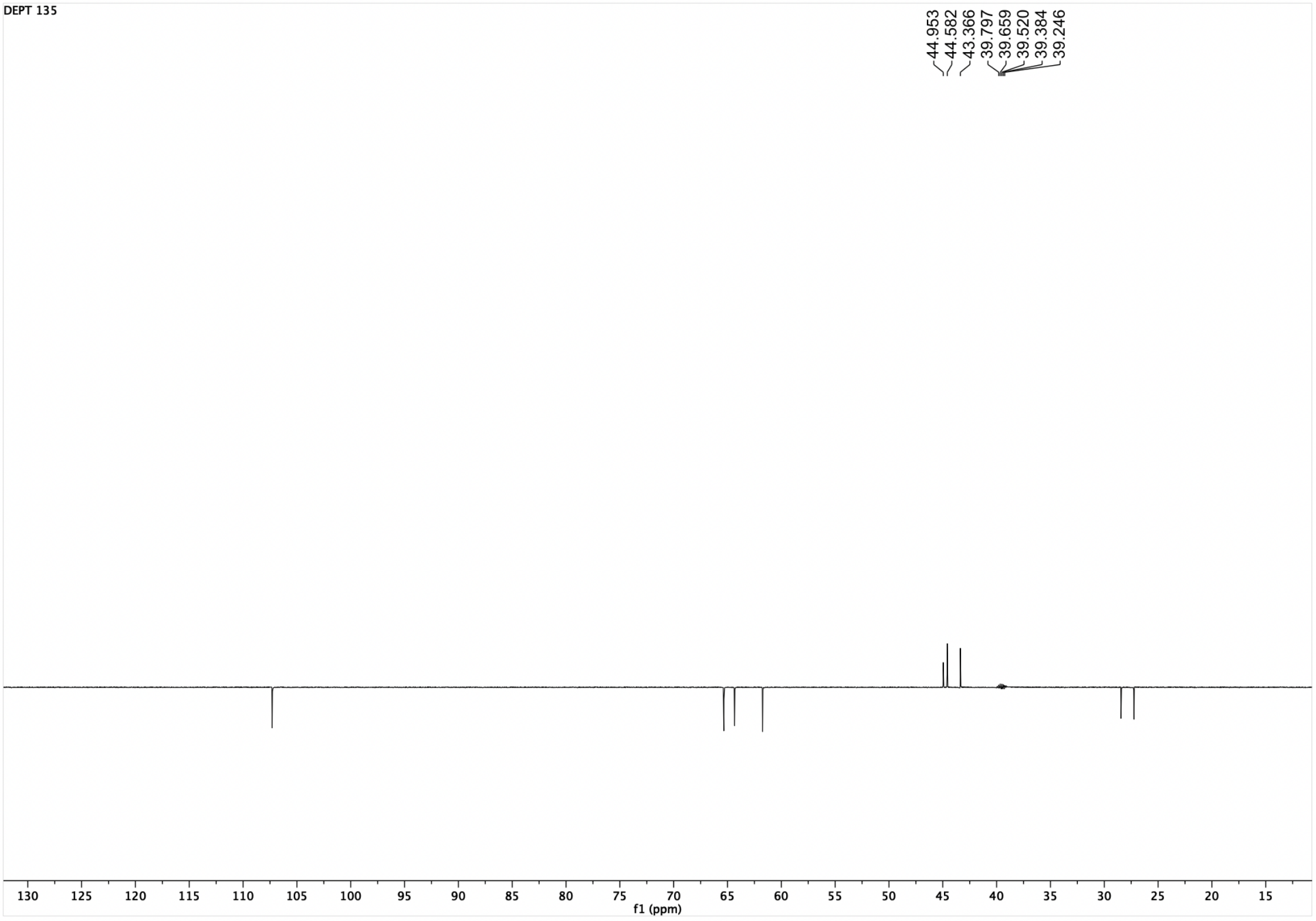

Figure S41. The DEPT 135 Spectrum of Compound **5** in DMSO-d_6

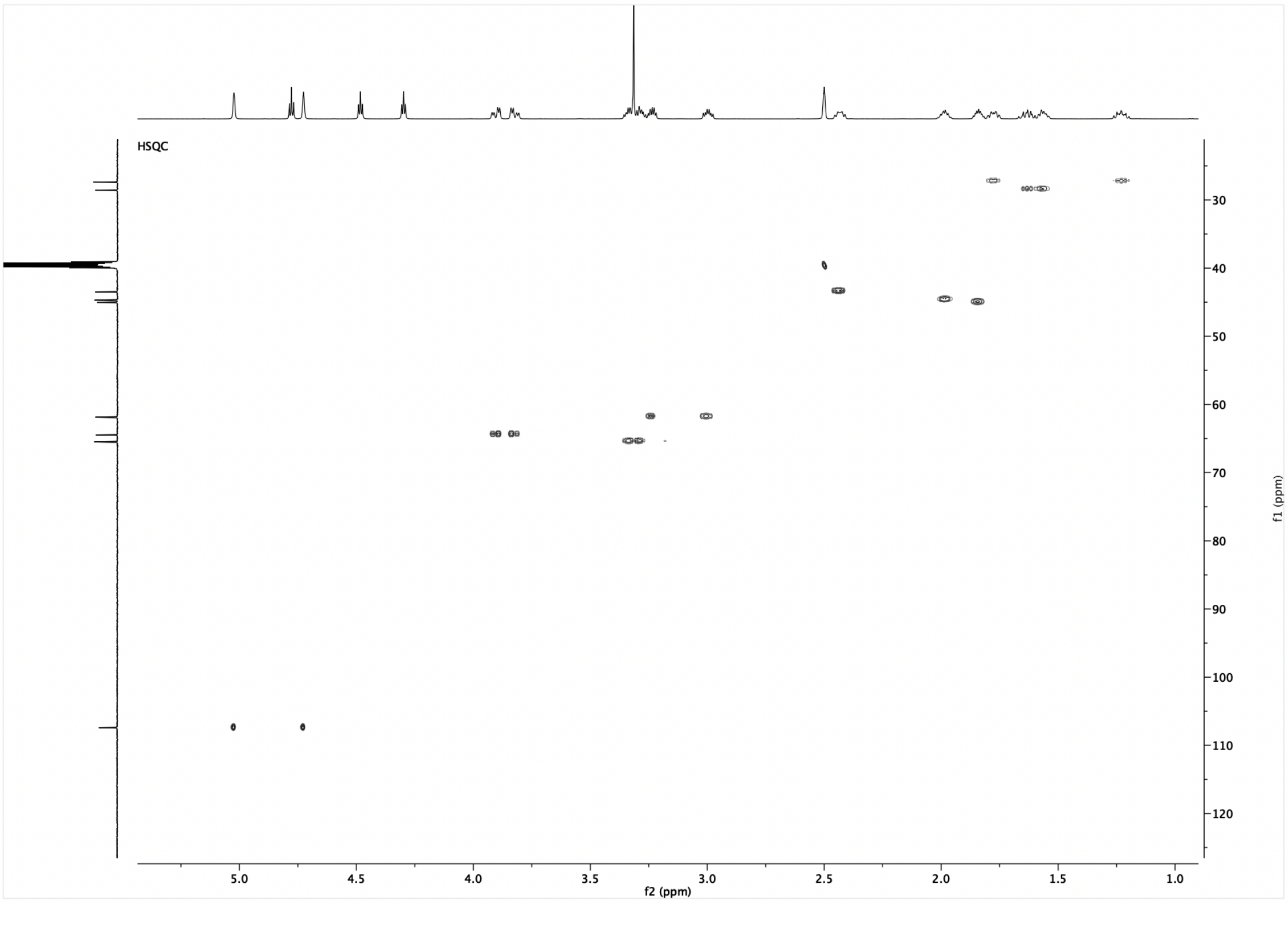

Figure S42. The HSQC Spectrum of Compound **5** in DMSO-d_6

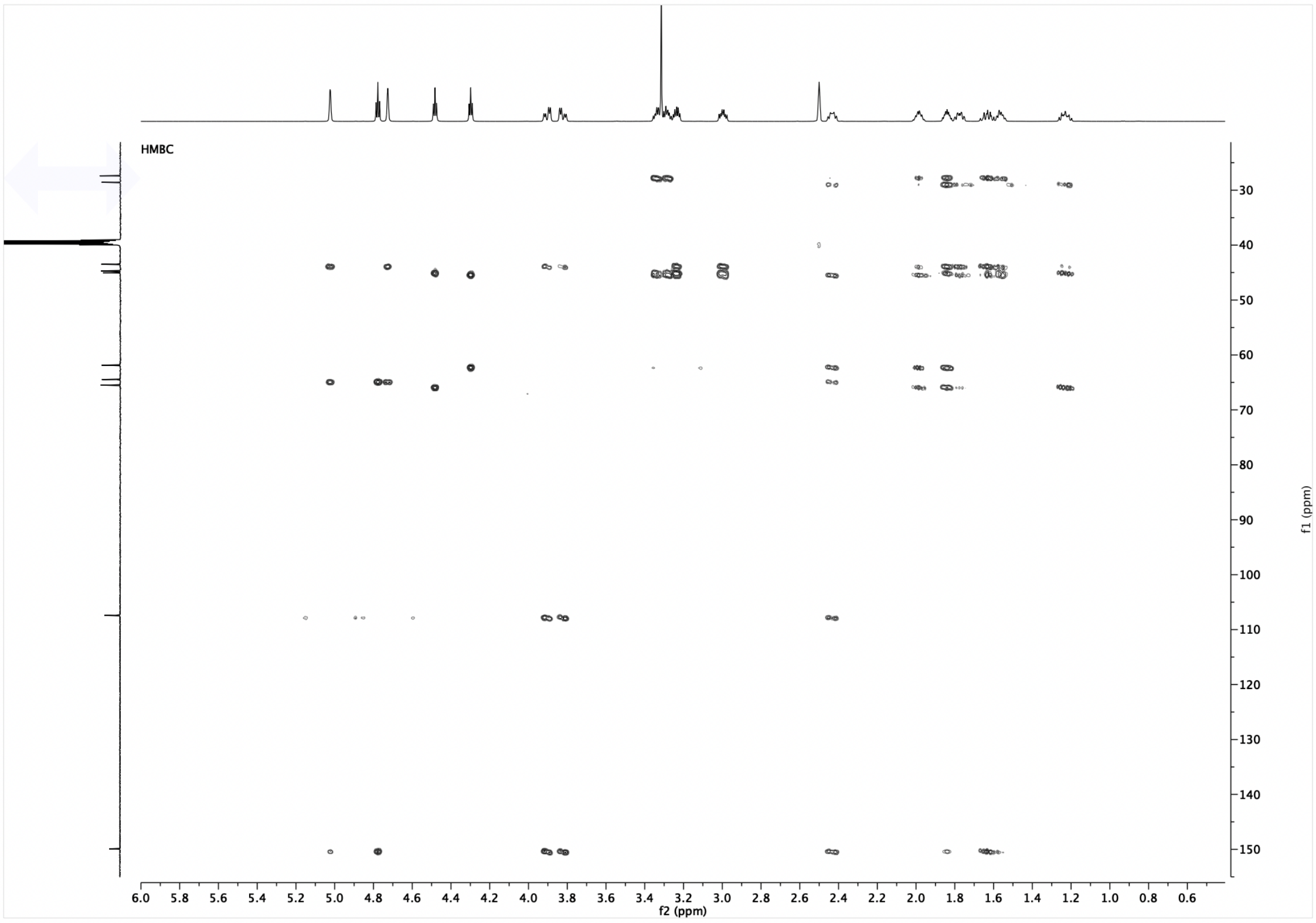

Figure S43. The HMBC Spectrum of Compound **5** in DMSO-d_6

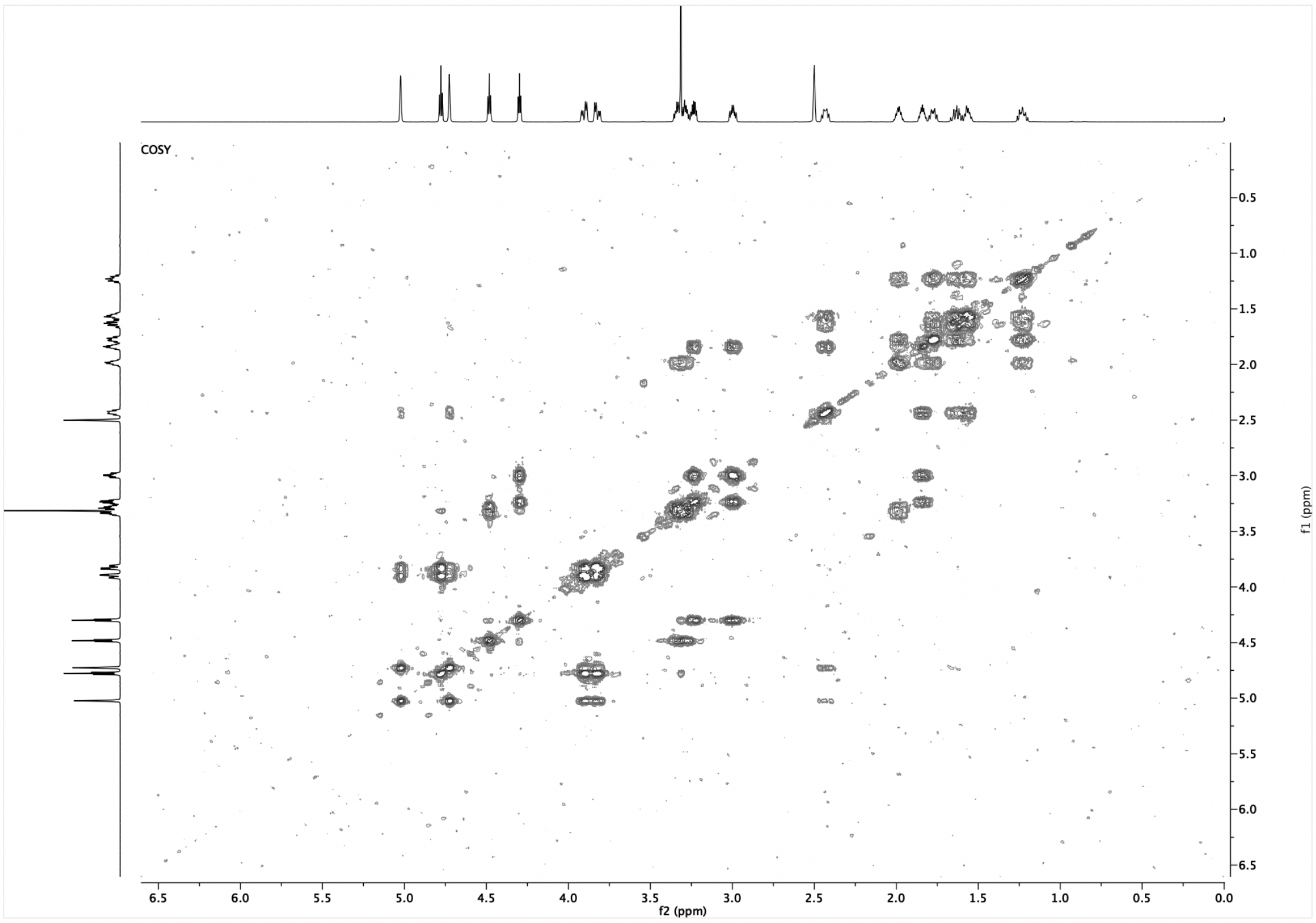

Figure S44. The ^{1}H-^{1}H COSY Spectrum of Compound **5** in DMSO-d_6

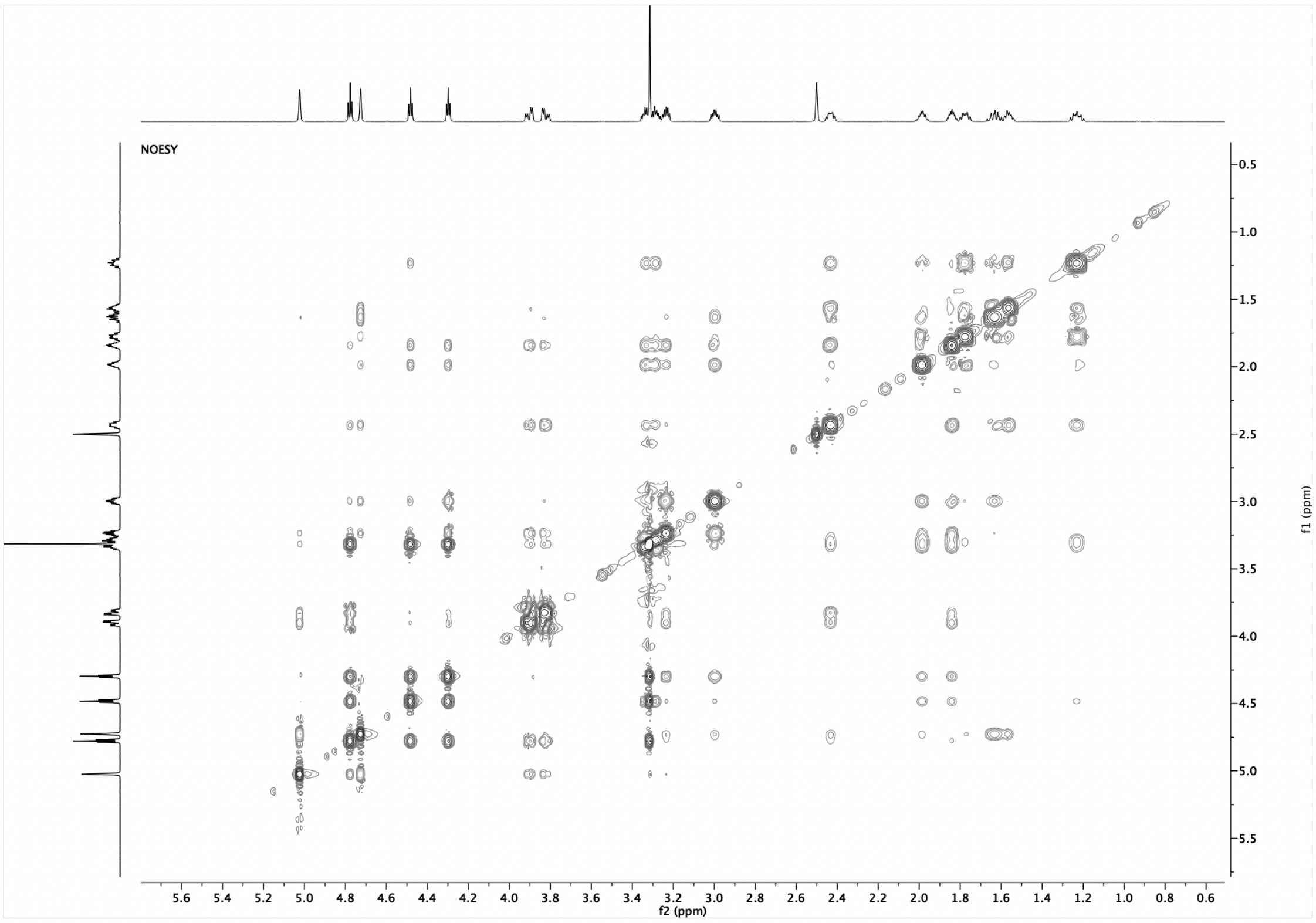

Figure S45. The NOESY Spectrum of Compound **5** in DMSO-d_6
